# TRANsCre-DIONE transdifferentiates scar-forming reactive astrocytes into functional motor neurons

**DOI:** 10.1038/s12276-026-01815-y

**Published:** 2026-09-11

**Authors:** Heeyoung An, Hye-Lan Lee, Doo-Wan Cho, Jinpyo Hong, Hye Yeong Lee, Jung Moo Lee, SunYeong Choi, In-Young Hwang, Junsung Woo, Jaekwang Lee, MinGu Park, Young-Su Yang, Su-Cheol Han, Yoon Ha, C. Justin Lee

**Affiliations:** 1 Center for Memory and Glioscience, Institute for Basic Science, Yuseong-gu, Daejeon, Republic of Korea; 2 Spine and Spinal Cord Institute, Department of Neurosurgery, College of Medicine, Yonsei University, Seoul, Republic of Korea; 3 Division of Jeonbuk Advanced Bio Research, Korea Institute of Toxicology, Jeongeup, South Korea; 4 Center for Glia-Neuron Interaction, Korea Institute of Science and Technology (KIST), Seoul, Republic of Korea; 5 Research Institute of Medical-Bio Convergence, Hallym University, Chuncheon, Republic of Korea; 6 Department of Biomedical Engineering, Ulsan National Institute of Science and Technology (UNIST), Ulsan, Republic of Korea; 7 Present Address: Laboratory Animal Resource & Research Center, Korea Research Institute of Bioscience and Biotechnology (KRIBB), Daejeon, Republic of Korea; 8 Present Address: Institute of Laboratory Animal Resource, Jeonbuk National University, Iksan, Republic of Korea

**Keywords:** Spinal cord injury, Neurodegeneration

## Abstract

In spinal cord injury (SCI), the scar-forming reactive astrocytes with upregulated glial fibrillary acidic protein (GFAP) proliferate aberrantly near the injury site, representing a potential cellular source for transdifferentiation into neurons to replenish dead neurons. However, the conventional use of *GFAP* promoter to target reactive astrocytes has two inherent problems: inadvertent conversion of normal astrocytes and low efficiency due to progressive weakening of promoter activity during transdifferentiation. Here, we present TRANsCre-DIONE, a dual-promoter split-Cre system combining *GFAP* and *Lcn2* regulatory elements with Cre-dependent *Neurog2* expression under the *EF1α* promoter, enabling selective targeting of scar-forming reactive astrocytes. This approach achieved 87% conversion efficiency and 96% specificity in vivo. After SCI, TRANsCre-DIONE caused transdifferentiation into Isl1-positive and ChAT-positive motor neurons, reduced astrogliosis, enhanced regeneration in surrounding cells, and a significant motor recovery. These findings suggest that TRANsCre-DIONE enables efficient and selective astrocyte-to-neuron conversion and represents a promising strategy for SCI repair.

**Figure:**
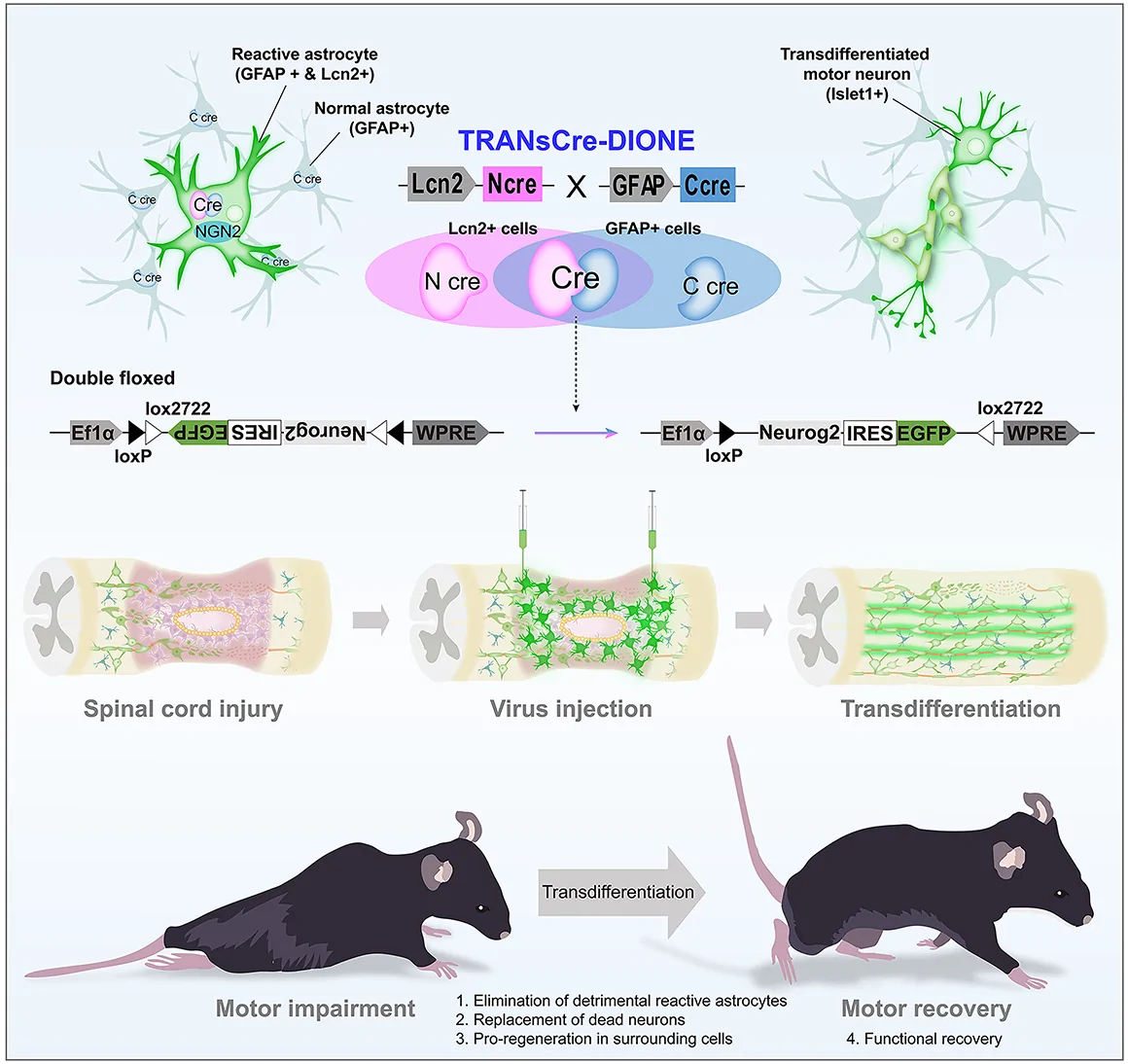

## Efficient astrocyte-to-neuron conversion enhances spinal recovery

Spinal cord injuries often lead to irreversible neuronal damage and limited treatment options, primarily focussing on symptom management. This study explores a novel approach to neuronal regeneration by transdifferentiating reactive astrocytes into neurons using a genetic tool called TRANsCre-DIONE. The method uses a split-Cre system with specific promoters to target reactive astrocytes, expressing the transcription factor Neurog2 to induce neuronal conversion. The study demonstrates that this approach can effectively convert reactive astrocytes into functional neurons in both mouse and monkey models, significantly improving motor function in spinal cord injury models. The findings highlight the potential of Neurog2 to generate region-specific neurons, such as GABAergic neurons in the striatum and motor neurons in the spinal cord, without tumorigenic risks. This research suggests a promising direction for future therapies targeting neuronal regeneration in spinal cord injuries and other neurodegenerative conditions. "This summary was initially drafted using artificial intelligence, then revised and fact-checked by the author."

## Introduction

Spinal cord injury (SCI) induces irreversible neuronal death and glial scar formation, which results in permanent motor and sensory dysfunction^1–3^. Although many attempts have been set forth to regenerate damaged spinal cord, currently doctors prescribe medications only to relieve pain to patients with SCI. Despite substantial efforts, therapeutic options remain largely limited to symptomatic management, and effective strategies for neuronal regeneration and functional restoration are still lacking^4–6^. The much-hoped-for stem cell therapy still has a long way to go in clinics owing to its issues with low efficacy and safety. The safety issues include tumor formation, immune rejection, and unnecessary pain by reprogrammed neurons forming improper neural connections^7,8^. These serious risks are majorly caused by transplantation of induced stem cells, which still might have pluripotency^9^.

To overcome the limitations of transplantation, the concept of transdifferentiation or direct reprogramming of one type of differentiated resident cells into other cell types has been proposed^9,10^. It has been reported that diverse cell types, including fibroblast, microglia, and astrocyte, can be transdifferentiated into neurons using various transcription factors^11–15^. Among these cell types, the glial fibrillary acidic protein (GFAP)-positive astrocytes are known as the most abundant cell type in the brain^16^. Under normal conditions, astrocytes provide various neurotrophic factors for neuronal growth and survival, regulate ionic homeostasis by taking up potassium ions and glutamate, and engage in synaptic transmission and plasticity by releasing gliotransmitters^17^. However, under pathological conditions such as in SCI, these normal astrocytes transform at the site of injury into reactive astrocytes and become hypertrophied and proliferative, especially under a severe condition^18^, to eventually form a glial scar^19–21^. The severely reactive astrocytes overexpress oxidizing enzymes such as MAO-B to cause oxidative stress and can adopt neurotoxic features under certain conditions^22,23^. Therefore, these proliferating and scar-forming severely reactive astrocytes represent a potential cellular substrate for transdifferentiation into neurons to remove these toxic severely reactive astrocytes and replace dead neurons after injury.

Nevertheless, recent studies have emphasized that reactive astrocytes are not a homogeneous population but display considerable heterogeneity, including both neurotoxic and protective subtypes. Indeed, glial scars — traditionally considered inhibitory to regeneration — also have protective roles by restricting lesion expansion and supporting tissue integrity^18,19^. Therefore, reprogramming strategies must carefully balance the selective targeting of deleterious reactive astrocyte subtypes while sparing reparative ones.

*GFAP* promoter has been widely used to both target astrocytes and drive expression of transcription factors for transdifferentiation into neurons^2,11,12,24,25^. Despite the universal application of the *GFAP* promoter^26^, the efficiency of transdifferentiation in vivo has been shown to be lower than 30% in a mouse model of SCI^2^. The low efficiency is likely due to premature termination of astrocyte transdifferentiation, caused by reduced expression of a transcription factor driven by the GFAP promoter, which itself is downregulated as astrocytes convert into neurons. Therefore, the use of *GFAP* promoter to drive the expression of a transcription factor in astrocytes for transdifferentiation may not be optimal. More importantly, using solely *GFAP* promoter is not an ideal strategy for targeting reactive astrocytes because GFAP is also expressed in some neural progenitor cells as well as normal astrocytes^27,28^. This raises a possibility that some neural progenitor cells and normal astrocytes might unintentionally transdifferentiate into neuron-like cells. In fact, although a recent study reported a high transdifferentiation efficiency of 80% with only *GFAP* promoter to target reactive astrocytes in the brain of Alzheimer disease and Huntington’s disease mouse models^11,29^, the apparent high efficiency might have been overestimated by an undesirable targeting of neural progenitor cells and normal astrocytes. Therefore, to selectively target reactive astrocytes while sparing neural progenitor cells and normal astrocytes, we reasoned that incorporating reactive astrocyte-associated promoters, such as *Lcn2* (ref. ^30^) and inducible nitric oxide synthase (iNOS)^31,32^, could improve targeting specificity. Moreover, to continuously drive the expression of a transcription factor even in the transdifferentiated state, it is necessary to utilize a strong universal promoter, whose activity is maintained high regardless of the cell types. To prevent an inadvertent expression of a transcription factor in untargeted cells under a strong universal promoter, the gene of transcription factor should remain in an inactive form, whereas it should be switched to an active form at once in targeted cells. To address these requirements, we have utilized a split-Cre system^33,34^ under two separate reactive astrocyte-specific promoters to activate the transcription factor via Cre/DIO (double-floxed inversed orientation)-dependent inversion^35^. The inverted gene of transcription factor is then continuously expressed as it is driven by a strong universal promoter *EF1α*^36^.

To transdifferentiate reactive astrocytes into motor neurons in SCI model, we have considered various transcription factors that have been used to transdifferentiate astrocytes into neurons. To transdifferentiate fibroblasts into neurons directly, achaete-scute family bHLH transcription factor 1 (*ASCL1*) has been included in combination with other transcription factors such as *Sox2, Oct4*, and *Dlx1* (ref. ^37^). To transdifferentiate astrocytes into neurons, *Neurog2* and its downstream factor, *NeuroD1* (ref. ^38^), have been sequentially introduced as a single transcription factor in vitro^12,39,40^ and in vivo^11,41,42^. To transdifferentiate fibroblasts into Isl1-positive motor neurons, *Neurog2* has been included in combination with other transcription factors^37,43^. However, *Neurog2* has not been used for transdifferentiation of reactive astrocytes into motor neurons in SCI model.

Although other transcription factors such as *NeuroD1* have been widely explored for astrocyte-to-neuron conversion, their application for specific generation of motor neurons in SCI remains limited^44^. Although a direct experimental comparison between Neurog2 and NeuroD1 was not performed in this study, our selection of Neurog2 represents a hypothesis-driven approach^11,12,38,39,45^. Given its robust association with motor neuron fate specification in prior developmental and reprogramming studies, we hypothesized that Neurog2 would be a highly effective candidate for replacing lost motor neurons. In addition to assessing conversion efficiency and cellular specificity, we further investigated the molecular and functional consequences of reprogramming. Transcriptomic profiling (RNA sequencing (RNA-seq)) was performed to characterize global gene expression changes associated with astrocyte-to-neuron conversion. Furthermore, chemogenetic modulation using DREADD was used to evaluate the functional contribution of induced neurons to behavioral recovery.

In this study, we use TRANsCre-DIONE (Transdifferentiation of Reactive Astrocytes into Neurons by split-Cre using GFAP and Lcn2 or iNOS turning on of DIO-Neurogenin2 under *EF1α* promoter), a unique combination of split-Cre under two separate promoters for reactive astrocytes and Cre/DIO-dependent inversion of *Neurog2* under a strong universal promoter of *EF1α* to transdifferentiate scar-forming reactive astrocytes into Isl1-positive motor neurons in hope for functional recovery in mouse model of SCI.

## Materials and methods

### Animals

#### Mouse

To differentiate reactive astrocytes into neurons directly in the brain, 7–10-week-old C57BL/6J mice (DBL; Daehan Bio Link, Korea) were used for immunohistochemistry (IHC) and electrophysiology experiments. Mice were housed in an animal facility permitted by the Association for Assessment and Accreditation of Laboratory Animal Care (AAALAC). All animals were maintained in a vivarium with light/dark cycle (8:00 AM–8:00 PM). Animal care and handling were performed according to the directives of the IACUC of KIST (Seoul, Republic of Korea), Yonsei University (Protocol No. 2016-0027; Seoul, Republic of Korea), and IBS (Daejeon, Republic of Korea). Animals were randomly used for all experiments.

#### Rat

In this study, SD rats (200 ± 20 g; OrientBio, Gyeonggi-do, Korea) were used and kept in an animal facility permitted by the AAALAC. All experimental procedures with rats were conducted following protocols approved by the IACUC of Yonsei University (Protocol No. 2016-0027; Seoul, Republic of Korea).

#### Monkey

Two-to-six-year old cynomolgus monkeys (*Macaca fascicularis*, 2 males) were purchased from NafoVanny (Dong Nai Province, Vietnam) and weighed 4.1 kg and 3.9 kg on the dosing day. The monkeys were housed individually in a stainless-steel cage (543 mm width × 715 mm depth × 818 mm height) and acclimatized in the study room after a 30-day quarantine period. Throughout the study period, supplements, toys, and stainless-steel mirrors were supplied for environmental enrichment. The room conditions were automatically controlled based on standard operating procedures (a temperature of 20–29 °C, a relative humidity of 30–70%, a lighting cycle of 12 h dark/12 h light at ≥300 lux, and 10–20 air changes per hour). Filtered, ultraviolet light-irradiated municipal tap water was allowed *ad libitum*, and 120 g of commercial monkey chow (Certified Primate Diet No. 5048, PMI Nutrition International, USA) was provided daily. The animals were identified using body tattoos and cage cards. This study was performed in facilities approved by the AAALAC International. All procedures were approved by the Institutional Animal Care and Use Committee (IACUC) of the Korea Institute of Toxicology.

### DNA cloning and virus packaging

cDNAs encoding for full-length *Lcn2* (GenBank accession no. NM_008491.1) and *Neurog2* (GenBank accession no. NM_008430) were obtained by using an RT-PCR-based cloning from mouse brain. *iNOS* promoter (NM_000625, Product ID: HPRM30585) was purchased from Genecopoeia. GFAP-Ccre and GFAP-Ncre vectors were kindly provided by R. Sprengel (Max Planck Institute for Medical Research). pAAV-GFAP-Cre, pAAV-GFAP::Ccre, pAAV-GFAP::Ncre, pAAV-Lcn2::Ncre, pAAV-iNOS::Ncre, pAAV-EF1α::DIO-Neurog2-IRES-GFP, pAAV-EF1α::DIO-GFP, and pAAV-EF1α::DIO-Neurog2-IRES-hM4Di (for chemogenetic modulation) were packaged into adeno-associated virus (AAV).

### Astrocytes primary culture

Primary cultured astrocytes were electroporated at 4 DIV with a mixture of three plasmids (pAAV-EF1α-df-Ngn2-IRES-GFP, pAAV-Lcn2-Ncre, pAAV-GFAP-Ccre, 1:1:1, 5 μg each), and astrocyte media were changed to neuronal media 4 days after gene transfection. Astrocyte medium contains 10% HI Horse serum (Gibco, 26050-088), 10% HI Fetal Bovine Serum (Gibco, 10082-147), 1% Penicillin–Streptomycin (Gibco, 15140-122) in Dulbecco's modified Eagle's medium (DMEM, Corning, 10-013-CVR), neuronal media inducing neuronal differentiation including 2% B27 (Invitrogen, 17504-044) and 2% Glutamax (Gibco, 35050-061) in F-12 media (Gibco, 11765-054).

### Stereotaxic surgery in brain

#### Mouse

Seven-to-ten-week-old C57BL/6 mice were used. Mice were anesthetized by intraperitoneal injection of 2% avertin (20 μl g^−1^) and placed into stereotaxic frames. AAV (titer 1.5 × 10^12^ GC) was loaded into a 22-gauge Hamilton syringe and injected bilaterally into the mouse striatum region (+0.5 mm AP, ±1.85 mm ML, −3.0 mm DV from the dura) at a rate of 0.2 μl min^−1^ (total 2 μl) with a 25 μl syringe using a syringe pump (KD Scientific, Holliston, MA, USA).

#### Monkey

Animals (age 2–6 years) were anesthetized with ~8–10 mg/kg of zoletil 50 (Virbac Korea) 30 min before being clamped to the stereotaxic instrument (David Kopf instrument, model 1404). An aliquot of 25 µl virus was injected using the Hamilton syringe and infusion pump at a speed of 2.5 µl/min. After surgery, monkeys were prescribed 20 mg/kg of cephalosporin-C twice a day for 5 days, 2 mg/kg of ketoprofen once a day for 3 days, and 1 mg/kg of prednisolone acetate once a day for 5 days. To prevent infection following surgery, surgical sites were disinfected with betadine for 5 days. A mixture of AAV (2/5)-iNOS-Ccre, AAV (2/5)-GFAP-Ncre, and AAV (2/5)-DF-GFP and a mixture of AAV (2/5)-iNOS-Ccre, AAV (2/5)-GFAP-Ncre, and AAV (2/5)-DF-NGN2-IRES-GFP were injected in the putamen (AP +1.8, ML +12.00, DV −5.00 from bregma).

### Establishment of SCI model and AAV injection

For the establishment of SCI model, C57BL/6 mice (20 ± 2 g; OrientBio) and Sprague-Dawley (200 ± 20 g; OrientBio) rats were used, housed in an animal facility permitted by the AAALAC. All animals were anesthetized with ketamine (100 mg/kg; Yuhan, Seoul, Republic of Korea), Rompun (10 mg/kg; Bayer Korea, Seoul, Republic of Korea), and Isotroy 100 (isoflurane, 1–3%; Troikaa Pharmaceuticals Ltd, Gujarat, India). Laminectomy was performed at thoracic number 10 to expose the spinal cord. The spinal cord was compressed for 3 s by self-closing forceps (Fine Science Tools, North Vancouver, Canada). After SCI, muscle and skin were closed using 6/0 non-absorbable nylon sutures. Sham animals were operated by laminectomy only. Spinal-cord-injured animals were randomly separated by phosphate-buffered saline (PBS), Ctrl, and Ngn2. At 2 weeks after injury, 1 μl PBS and AAV were injected at two locations (1 mm proximal and distal to the injured site) using a 33-gauge Hamilton syringe. The injection rate was constantly maintained at 0.2 μl/min. After injection, the needle was kept at the injection site for additional 5 min. To assess the cell-type specificity of GFP-expressing cells, co-immunostaining for Iba1 or Lcn2 with GFAP was performed (Supplementary Fig. 2). The SCI model and viral injections into the spinal cord were conducted as described earlier. Mice were sacrificed 3 weeks after viral injection, and spinal cord tissues were collected for IHC.

### BrdU assay

For detection of cell proliferation, BrdU (50 mg/kg; Sigma-Aldrich, B5002) was administered by intraperitoneal injection. BrdU was injected daily for 7 days, starting at 2 weeks and again at 7 weeks after injury. BrdU-injected mice were sacrificed at the last day of BrdU injection. The concentration of BrdU solution was 10 mg/ml in 0.9% saline and filtrated before being injected to the mouse. BrdU was detected by IHC using the mouse anti-BrdU antibody (Thermo Fisher, ZBU30; 1:2000).

### Behavioral test

To confirm locomotor recovery, the behavioral testing was performed weekly from 1 to 8 weeks after injury in mice and from 1 to 10 weeks in rats. For measuring the movements of injured mice, the Basso mouse scale (BMS) was used to quantify the movement of hind limb during open field locomotion. The BMS score is categorized as 0 to 9. For BMS assessment following chemogenetic inhibition, clozapine *N*-oxide was administered 30 min before BMS measurement. A score of 0 indicates complete paralysis and a score of 9 indicates normal locomotion. To quantify movement of SCI rats, Basso, Beattie, and Bresnahan (BBB) motor scores were measured. BBB scoring includes three-joint movement-angle ranges, weighted stepping, gait coordination, and tail movement and has a range of 0–21. A score of 0 indicates complete paralysis and a score of 21 indicates normal movement. BMS and BBB scoring were performed by two different investigators blinded to animal grouping.

### Tissue sample preparation for Eriochrome Cyanine staining and IHC

For isolation of spinal cord, animals were sacrificed at 3 weeks and 8 weeks after injury. Blood was completely removed by saline perfusion and fixed by 4% paraformaldehyde (PFA, Duksan, Ansan, Korea). The isolated tissues were fixed in 4% PFA overnight and transferred to 30% sucrose for dehydration at 4 °C. Dehydrated tissues were embedded in optimal cutting temperature compound (Sakura Finetek, Torrance, CA, USA), frozen at −80 °C, and cut into a thickness of 20 μm by cryostat.

### EC staining

The sectioned tissues were dried at room temperature (RT) for 2 h and immersed in acetone for 5 min. After immersion, tissues were incubated at RT for 10 min and stained by EC solution (MERCK, Kenilworth, NJ, USA) at RT for 30 min. The stained tissues were rinsed by running water and 5% iron alum (Sigma-Aldrich, F3629) until the gray matter is observed. Tissues were differentiated by borax–ferricyanide solution (borax, Sigma-Aldrich, 71997; potassium ferricyanide, Sigma-Aldrich, 702587) and dehydrated by graded ethanol solutions of 70%, 90%, and 100%. After all processes, tissues were mounted by permanent mounting medium (Fisher Scientific, Hampton, NH, USA) and observed using a light microscope (DM 2500, Leica, Wetzlar, Germany).

### Immunohistochemistry

#### Immunohistochemistry in brain

Animals were euthanized at the end of the treatment period by an intravenous overdose of thiopental sodium, followed by blood collection via the posterior vena cava. They were fasted over 16 h before the necropsy. The animals were examined carefully for external abnormalities. The abdominal, thoracic, and cranial cavities were examined for abnormalities and the brain then removed and examined. Adult mice were deeply anesthetized with 2% avertin (20 µg/g) and perfused with 0.1 M PBS followed by ice cold 4% PFA. Excised brains were post-fixed overnight in 4% PFA at 4 °C and immersed in 30% sucrose for 24 h for cryo-protection. The 30–50 µm coronal sections were prepared with cryo-stat microtome (HM525, Thermo Fisher Scientific). Sections were rinsed in PBS three times and incubated for 1 h at RT with blocking solution (0.3% Triton X-100, 2% normal serum in 0.1 M PBS). Sections were incubated overnight in a mixture of primary antibodies with blocking solution at 4 °C on shaker. After washing three times with PBS, sections were incubated with corresponding secondary antibodies for 2 h and then rinsed three times with PBS. If needed, 4′,6-diamidino-2-phenylindole (DAPI) staining was added during the second wash step (Pierce, 1:3000). After being dried, stained tissues were mounted with an anti-fade mounting medium (Dako). A series of fluorescence images were obtained with a confocal microscope (Nikon, A1R), and images were processed for later analysis using ImageJ program imaging software.

#### Immunohistochemistry in spinal cord

The tissue sections were washed three times by 0.3% Tween 20 (Sigma-Aldrich, P1379) in PBS at RT for 5 min each and blocked by 10% normal donkey serum (Jackson ImmunoResearch, West Grove, PA, USA) in PBS containing 0.3% Triton X-100 (Sigma-Aldrich, X100) at RT for 1 h. After then, primary antibodies were treated such as goat anti-GFP (Abcam, Cambridge, UK, ab6662, 1:1000), chicken anti-GFAP (Abcam, ab4674, 1:2000), chicken anti-MBP (Abcam, ab134018, 1:400), rabbit anti-MAP2 (Abcam, ab32454, 1:1000), rabbit anti-NeuN (Abcam, ab177487, 1:800), mouse anti-islet1 (DSHB Iowa City, IA, USA, 39.4D5, 1:100) and goat anti-ChAT (MilliporeSigma, AB144P, 1:500), and rabbit anti-Ki67 (Abcam, ab15580, 1:500) and incubated at 4 °C overnight. After washing three times with 0.3% Tween 20, sections were incubated with species-specific secondary antibodies conjugated with fluorescent agents such as Cy^TM^3-donkey anti-mouse IgG (H + L) (Jackson ImmunoResearch, 175-165-151; 1:600), Cy^TM^3-donkey anti-chicken IgG (H + L) (Jackson ImmunoResearch, 175-165-155; 1:600), Alexa Fluor 647-donkey anti-rabbit IgG (H + L) (Jackson ImmunoResearch, 711-606-152; 1:300), Cy^TM^5-donkey anti-chicken IgG (H + L) (Jackson ImmunoResearch, 703-175-155; 1:300), and DyLight^TM^405-donkey anti-chicken IgY_++_ (H + L) (Jackson ImmunoResearch, 703-476-155; 1:600) at RT for 1 h. Incubated sections were washed three times using 0.3% Tween 20 and stained with DAPI (DAPI; Vector Laboratories, Peterborough, UK). The fluorescence was observed under a confocal laser scanning microscope (LSM700, Carl Zeiss, Oberkochen, Germany).

### Electrophysiology

#### Slice preparation

Coronal mouse brain slices (300 μm) containing the striatum were acutely prepared from C57BL/6 (age 7–10 weeks). Coronal sections of the spinal cord from mice and rats were sliced at thicknesses of 300 µm and 400 µm each, while submerged in chilled artificial cerebrospinal fluid (ACSF). Following decapitation, the brain was rapidly removed and placed in cold ACSF having the following composition (in mM): 130 NaCl, 24 NaHCO_3_, 3.5 KCl, 1.25 NaH_2_PO_4_, 1 CaCl_2_, 3 MgCl_2_, and 10 glucose, pH 7.4; RT with oxygenation (95% O_2_ and 5% CO_2_). The slices were made using an oscillating tissue slicer (DSK LinearSlicer, Kyoto, Japan) at 4 ^o^C and stored at RT with oxygenation (95% O_2_ and 5% CO_2_), and prepared slices were left to recover for at least 1 h before recording.

#### Ex vivo patch-clamp recording

Each slice that was studied was transferred from a recovery/holding reservoir to the recording chamber of a fixed-stage upright microscope (Olympus) and submerged in oxygenated ACSF that was supplied to the chamber at a rate of 1.5–2 ml/min. The submerged slice was visualized either directly via the microscope’s optics or indirectly via a high-resolution CCD camera system (Orca Flash 2.1, Hamamatsu) that received the output of a CCD camera attached to the microscope’s video port. Experiments with a holding current of more than −100 pA or in which there was a change in input resistance >30% of the control were rejected. Recordings were obtained using Multiclamp 700A amplifier (Molecular Devices, Sunnyvale, CA, USA) and were filtered at 2 kHz. Current recordings under ramp protocol and step were digitized at 10 kHz with DigiDATA 1550B (Molecular Devices) and analyzed using pCLAMP 10 software (Molecular Devices). Whole-cell recordings from GFP-expressing cells were carried out with internal solution composed of (mM): 120 potassium gluconate, 10 KCl, 1 MgCl_2_, 0.5 EGTA, 40 HEPES (pH 7.2 was adjusted with KOH).

### Illumina HiSeq library preparation and sequencing

Spinal cord tissues were collected from two groups: SCI/Ctrl and SCI/Neurog2 (*n* = 3 per group). Total RNA was extracted from each sample and used for library preparation. Sample libraries were prepared using the NEBNext Ultra RNA Library Prep Kit (NEB, no. E7530), Multiplex Oligos for Illumina (NEB, no. E7335), and the Poly(A) mRNA Magnetic Isolation Module (NEB, no. E7490), according to the manufacturer’s instructions. Detailed protocols are available on the manufacturer’s website. Library quality and concentration were assessed using the Agilent 2100 Bioanalyzer with the High Sensitivity DNA kit (Agilent Technologies). Libraries were prepared for sequencing following the Illumina HiSeq reagent kit preparation guidelines. Briefly, pooled libraries were diluted to 2 nM, denatured with 0.2 N NaOH, and further diluted to 20 pM using Illumina HT1 buffer. The libraries (600 μl) were loaded onto a 150-cycle (2 × 75 bp paired-end) reagent cartridge and sequenced on an Illumina HiSeq platform. After sequencing, base calling and demultiplexing were performed to generate FASTQ files for downstream analysis.

### NGS data analysis

Raw BCL files generated from the Illumina HiSeq platform were converted to FASTQ format and demultiplexed based on index sequences. The resulting FASTQ files were imported into Partek Genomics Suite (Flow version 10.0.21.0328; Partek Inc., St Louis, MO, USA) for downstream analysis. Read quality was assessed using FastQC. High-quality reads were aligned to the mouse reference genome (mm10, Ensembl release 99) using STAR (version 2.7.3a). Gene-level quantification was performed, and count data were normalized using the median ratio method implemented in DESeq2. Differential gene expression analysis was conducted between SCI/Ctrl and SCI/Neurog2 groups (*n* = 3 per group) using DESeq2. Pathway enrichment analysis was performed based on the Gene Ontology (GO) database.

### Statistics

The significance of data for comparison was assessed by Student’s two-tailed unpaired *t* test or ordinary one-way analysis of variance. Non-parametric tests (Mann–Whitney test, Kruskal–Wallis test with multiple comparisons, and Dunnett's multiple comparison test) were used where indicated in the figure legends. Data are presented as mean ± SEM. Levels of statistical significance are indicated as follows: *P < 0.05, **P < 0.01, and ***P < 0.001.

## Results

### Genetic strategy of transdifferentiation in the brain of a mouse and non-human primate

**Fig. 1:**
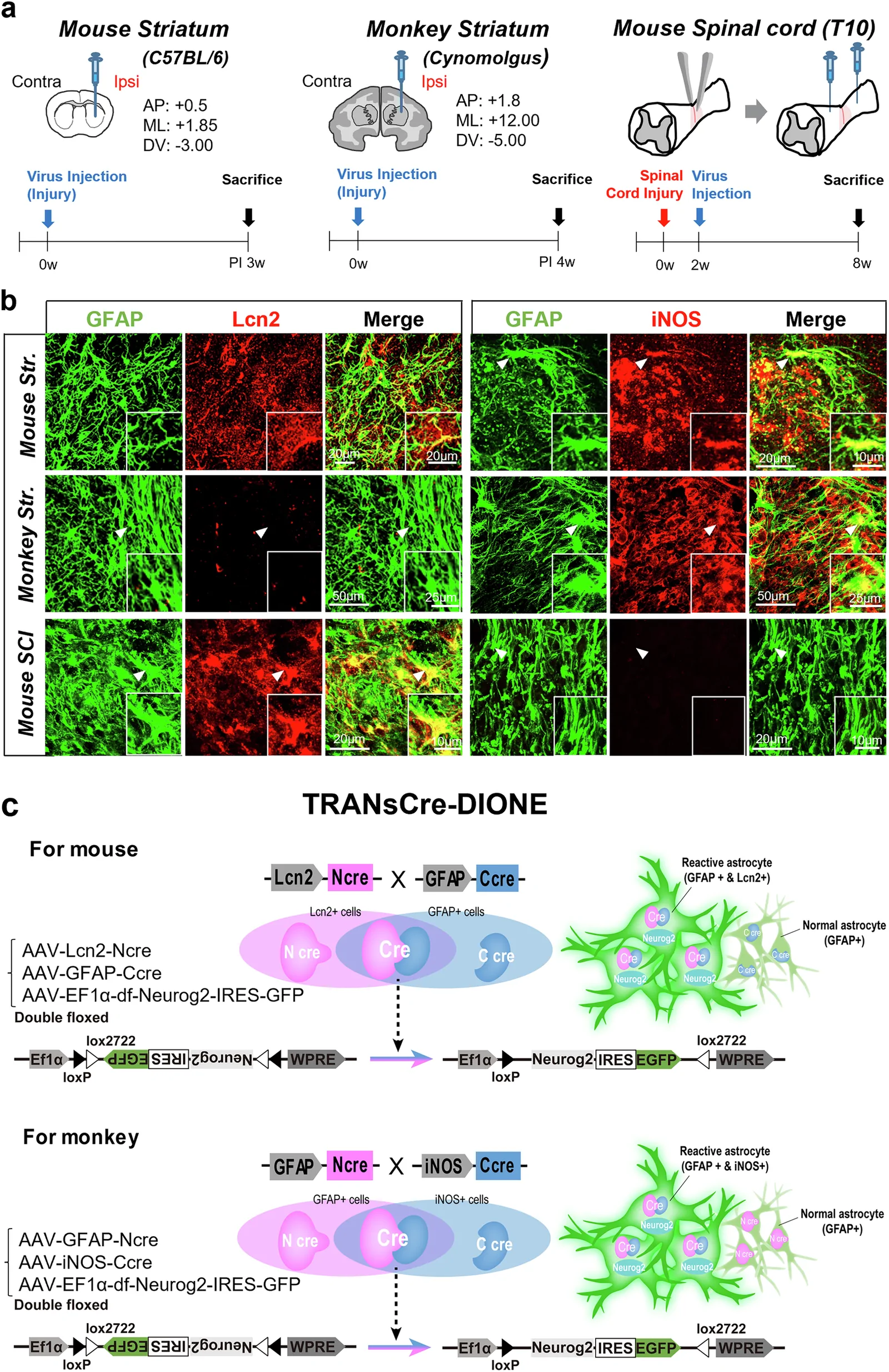
The genetic strategy of transdifferentiation in the brain of a mouse and non-human primate. **a** Experimental protocol for induction of reactiveness by virus injection and the coordination of the surgery in the striatum of the mouse (left), cynomolgus monkey (middle), and the mouse spinal cord (right). Unilateral injection was performed in striatum of mice (AP: +0.5, ML: 1.85, DV: −3.00) and monkey (AP: +1.8, ML: 12.00, DV: −5.00). Injury was given at the level of Thoracic 10 of mouse spinal cord with self-closing forceps. Virus was injected into two points beside the lesion site 2 weeks after the injury. Mice were sacrificed post-injection (PI) 3 weeks. Monkeys were sacrificed PI 4 weeks. Spinal cord injury (SCI) mice were sacrificed post-injury 8 weeks (PI 6 weeks). **b** Lcn2 (red, left panel) and inducible nitric oxide synthase (iNOS) (red, right panel) expression and colocalization with GFAP (green, both) in the injured striatum (Str.) of mouse and monkey and mouse SCI (arrowhead: colocalization of GFAP and Lcn2, or GFAP and iNOS). Scale bar, 20 μm (mouse Str. and mouse SCI), 50 μm (monkey Str.). **c** TRANsCre-DIONE, the strategy for transdifferentiation by expression of *Neurog2* selectively in reactive astrocytes of mouse brain (upper) and monkey brain (lower). AAV adeno-associated virus, AP anteroposterior, ML mediolateral, DV dorsoventral, PI post-injection.

The scar-forming reactive astrocytes exhibit upregulation of *GFAP* and neuroinflammation-related genes such as lipocalin2 (*Lcn2*) and iNOS^31,32^. To express the transcription factor in reactive astrocytes more specifically, we introduced split-Cre, which is the split form of the whole improved Cre (iCre), divided into two parts composed of N-terminal part of the iCre (Ncre) and C-terminal part of the iCre (Ccre)^33,34^, under two different promoters. To confirm the induction of the gene products of *Lcn2* and *iNOS* in the reactive astrocytes, we immunostained the injured tissues with antibodies against Lcn2 and iNOS. To apply the traumatic injury to the brain of the mouse and monkey, the 32-gauge and 23-gauge needle was used for virus injection, respectively (Fig. 1a). To induce an injury to the mouse spinal cord, a compression injury was introduced by self-closing forceps for 3 s followed by virus injections into the two points next to the injury site (Fig. 1a). The GFAP signal increased in both mouse and monkey when the tissues were injured (Supplementary Fig. 1). Lcn2 was induced in the injured mouse brain and spinal cord (Fig. 1b), whereas only the iNOS signal was induced without the Lcn2 signal in the injured monkey’s brain tissues (Fig. 1b). Lcn2 and iNOS were never observed in the uninjured tissues (Supplementary Fig. 1). Along with these results, for gene expression in reactive astrocytes specifically, *Lcn2* was used with *GFAP* as promoters of split-Cre in the mouse brain and spinal cord, and *iNOS* and *GFAP* were used for the promoters of split-Cre in the monkey’s brain (Fig. 1c). All genes were cloned and packaged into AAV for broad and robust gene expression in the brain and spinal cord. *Neurog2* was expressed only in reactive astrocytes because both sides of the split-Cre can be expressed only in reactive astrocytes to make a functional Cre. Thus, we used TRANsCre-DIONE, composed of pAAV-EF1α::DIO-Neurog2-IRES-GFP (DIO-Neurog2) with pAAV-Lcn2::NCre and pAAV-GFAP::CCre in the mouse and pAAV-iNOS::CCre and pAAV-GFAP::NCre in the monkey brain (Fig. 1c). In addition, GFP fluorescence (under IRES (internal ribosome entry site)) was used as a reporter for easy monitoring of TRANsCre-DIONE-containing cells, that is, untransdifferentiated reactive astrocytes or transdifferentiated neurons. The split-Cre system did not show any leakiness in the NCre or CCre only condition (Supplementary Fig. 3). With these results, we designed TRANsCre-DIONE using *Lcn2* and *GFAP* promoters of split-Cre for mouse and *iNOS* and *GFAP* promoters of split-Cre for monkey to express *Neurog2* in reactive astrocytes specifically.

### Transdifferentiation of reactive astrocytes into functional neurons in the mouse brain

**Fig. 2:**
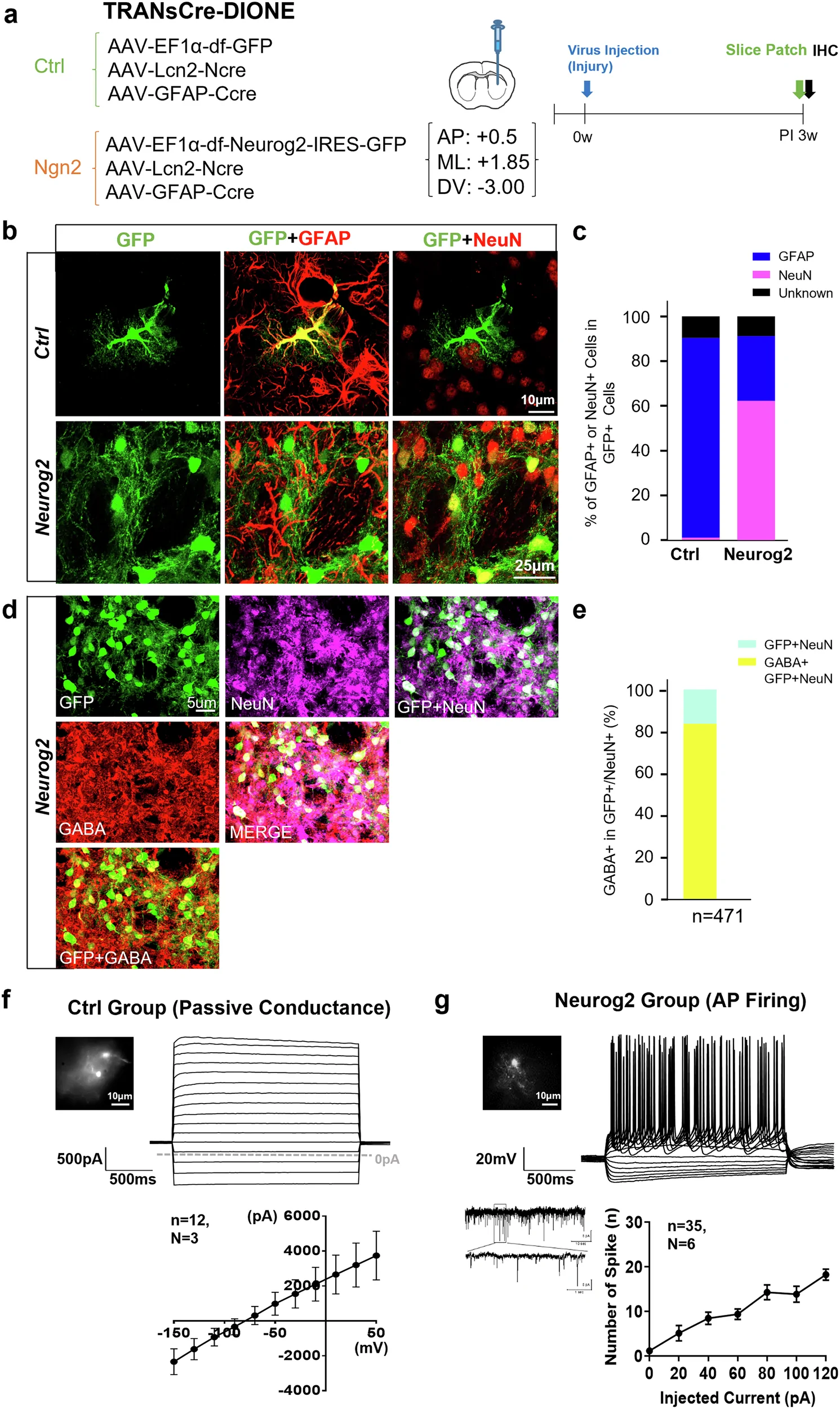
Transdifferentiation of reactive astrocytes into functional neurons in the mouse brain. **a** A mixture of three viruses (TRANsCre-DIONE, 2 μl of mixture) was injected into the striatum (AP: +0.5, ML: +1.85, DV: −3.0; bilaterally). The mice were sacrificed PI 3 weeks for immunohistochemistry (IHC) and ex vivo patch-clamp recording. **b** Colocalization of GFAP (red, middle column) and NeuN (red, right column) with GFP fluorescence (green, left column) in Ctrl (upper) and Neurog2 (lower) group. Scale bar, 10 μm (Ctrl group), 25 μm (Neurog2 group). **c** Stacked bar graph showing the percentage of GFAP (blue)-positive and NeuN (pink)-positive cells in GFP-expressing cells (black: undefined cells). *n* = 93 (Ctrl group), *n* = 135 (Neurog2 group). **d** Colocalization of GABA (red, middle column) and NeuN (magenta, upper) with GFP fluorescence (green) in Neurog2 group. Scale bar, 5 μm. **e** Stacked bar graph showing the number and percentage of GABA-positive (yellow) cells in NeuN-positive and GFP-expressing (cyan) cells. Total *n* = 471 in Neurog2 group. **f** Representative trace of passive conductance (Upper) recorded from GFP-expressing cells (inset, scale bar, 10 μm) in the control group by voltage-clamp step protocol. Scale bar, 500 pA, 500 ms. Averaged amplitude reached to around 4000 pA at 50 mV. Averaged *I*–*V* graph (lower) from voltage of −150 mV to 50 mV. *N* = 3, *n* = 12. **g** Representative action potential firing (upper) and the trace of spontaneous excitatory postsynaptic currents (lower, left) from GFP-expressing cells (inset, scale bar, 10 μm) in the Neurog2 group. The average number of spikes (lower, right) was increased by injected current. *N* = 6, *n* = 35. AAV adeno-associated virus, IHC immunohistochemistry.

To examine whether reactive astrocytes can specifically transdifferentiate into neurons in vivo upon expression of a transcription factor and to quantify the efficiency of this transdifferentiation, we injected TRANsCre-DIONE, a mixture of packaged AAV (1:1:1) for control group (AAV- EF1α-DIO-EGFP with AAV-Lcn2-NCre and AAV-GFAP-CCre) and Neurog2 group (AAV-EF1α-DIO-Neurog2-IRES-EGFP with AAV-Lcn2-NCre and AAV-GFAP-CCre) bilaterally into the mouse striatum (ML 1.85 mm, AP 0.5 mm, DV −3.0 mm). The mice were sacrificed 3 weeks after injection (Fig. 2a). We immunostained the tissues with antibodies against GFAP and NeuN for distinguishing the cell types of GFP-expressing cells. The efficiency of transdifferentiation was calculated by counting the number of GFAP-positive and NeuN-positive cells in GFP-expressing cells. Most of the GFP-expressing cells (62%, 84 out of 135 cells) were NeuN-positive in the Neurog2 group whereas only 1% (1 out of 93 cells) of GFP expressing cells were NeuN-positive in the control group (Fig. 2b, c). Most of the GFP-expressing cells (89%, 83 out of 93 cells) in the control group remained GFAP-positive astrocytes, whereas 28% (39 out of 135 cells) of the GFP-expressing cells in Neurog2 group were GFAP-positive (Fig. 2b, c). These results demonstrate that GFAP-positive reactive astrocytes transdifferentiate into neurons in the mouse striatum by expressing *Neurog2* solely.

Striatal neurons are predominantly GABAergic: ~95% are medium spiny neurons that co-express GABA and DARPP-32, whereas the remaining ~5% comprise aspiny GABAergic interneurons^44^. By contrast, it has been reported that the transdifferentiated neurons from cultured astrocytes upon overexpression of *Neurog2* were mostly glutamatergic neurons^12^, raising a strong possibility that the transdifferentiated neurons in the striatum are glutamatergic rather than GABAergic. To determine whether the transdifferentiated neurons in the striatum are GABAergic, we performed double-immunostaining with antibodies against NeuN and GABA (Fig. 2d). By surprise, we found that 83.64% of the NeuN+ GFP-expressing cells were GABA+ (Fig. 2e), indicating that the majority of the transdifferentiated neurons were GABAergic in the striatum. These results suggest that overexpression of *Neurog2* causes transdifferentiation into GABAergic neurons in the specific environment surrounded by GABAergic neurons.

To test the functional property of transdifferentiated neurons from reactive astrocytes by forced *Neurog2* expression, we performed whole-cell patch-clamp recording in the infected cells. GFP-expressing cells (Fig. 2f, inset) in the control group showed a large and linear passive conductance current (Fig. 2f, upper panel), which is a unique physiological property of astrocytes in the striatum (Fig. 2f). Contrary to the control group, GFP-expressing cells (Fig. 2f, inset) from the Neurog2 group showed robust action potential firing (Fig. 2g, upper panel) by the current-clamp recording with whole-cell patch clamp ex vivo. The spontaneous excitatory postsynaptic currents (EPSCs) (Fig. 2g, inset) and large sodium and potassium currents were also observed (Supplementary Fig. 5), indicating the maturity of the transdifferentiated neurons (Fig. 2g). The similar morphological change and action potential firing were observed within 14 days after transducing *Neurog2* in culture (Supplementary Fig. 4a–c). These results demonstrate that reactive astrocytes transdifferentiate into functional neurons by expressing solely *Neurog2* in reactive astrocytes.

### Transdifferentiation of reactive astrocytes into functional neurons in the monkey’s brain

**Fig. 3:**
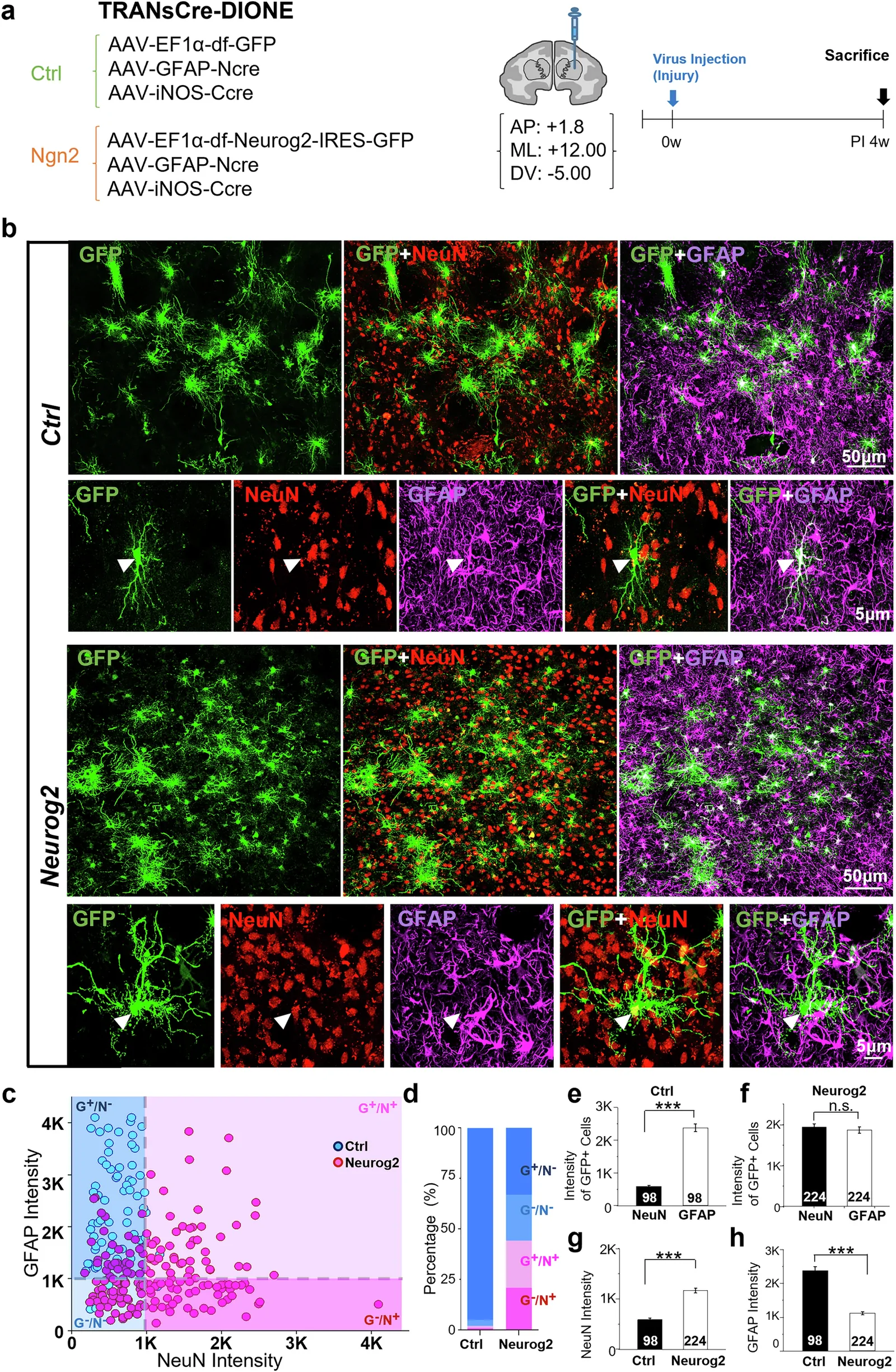
Transdifferentiation of reactive astrocytes into functional neurons in the monkey’s brain. **a** A mixture of three viruses (TRANsCre-DIONE, 25 μl of the mixture) was injected into the striatum (AP: +1.8, ML: +12.00, DV: −5.0; unilaterally). The monkeys were sacrificed PI 4 weeks for IHC. **b** Colocalization of GFAP (magenta) and NeuN (red) signal in GFP-expressing cells (green) in the control group (Upper) and Neurog2 group (lower). Scale bar, 50 μm (low magnification) and 5 μm (magnified images). Arrowhead means the colocalization of GFP and GFAP in the Ctrl group, or GFP and NeuN in the Neurog2 group. **c** Scatter plot of all GFP-expressing cells in Ctrl (blue dot) and Neurog2 (pink dot) groups classified as GFAP and NeuN intensity. Quadrant 1 (light pink), GFAP+/NeuN+ (G+/N+); quadrant 2 (blue), GFAP+/NeuN− (G+/N−); quadrant 3 (light blue), GFAP−/NeuN− (G−/N−); quadrant 4 (pink), GFAP−/NeuN+(G−/N+) (reference lines are drawn for dividing + or − at 1 K of both intensities). *n* = 98 (Ctrl group), *n* = 224 (Neurog2 group). **d** Stacked bar graph of the percentage of G+/N−, G−/N−, G+/N+, and G−/N+ in Ctrl and Neurog2 groups. **e**, Comparison of NeuN and GFAP intensity of GFP-expressing cells in the Ctrl group. Unpaired Student's *t* test (n.s., *P* > 0.05, *P < 0.05, **P < 0.01, ***P < 0.001, ****P < 0.0001). *n* = 98 (Ctrl group). **f** Comparison of NeuN and GFAP intensity of GFP+ cells in Neurog2 group. Unpaired Student's *t* test. *n* = 224 (Neurog2 group). **g** Comparison of NeuN intensity of GFP+ cells in Ctrl and Neurog2 groups. Unpaired Student's *t* test. *n* = 98 (Ctrl group), *n* = 224 (Neurog2 group). **h** Comparison of GFAP intensity of GFP+ cells in the Ctrl and Neurog2 groups. Unpaired Student's *t* test. *n* = 98 (Ctrl group) and *n* = 224 (Neurog2 group).

To investigate whether transdifferentiation occurs in the brain of non-human primates, we injected TRANsCre-DIONE into the putamen of cynomolgus monkeys (*Macaca fascicularis*) as described previously^46^ and harvested the brain tissues after 4 weeks (Fig. 3a). As mentioned earlier (Fig. 1c), *iNOS* was used as a promoter instead of *Lcn2* with *GFAP* for transducing *Neurog2* into reactive astrocytes in the monkey’s brain (Fig. 3a). Harvested brain tissues were immunostained with antibodies against GFAP and NeuN as well (Fig. 3b). GFP-expressing cells rarely colocalized with NeuN in the control group, while considerable GFP-expressing cells were NeuN-positive in the Neurog2 group (Fig. 3b). These results demonstrate that the reactive astrocytes in the monkey’s brain also transdifferentiate into NeuN-positive neurons by expressing *Neurog2*. To investigate whether the infected cells completely transdifferentiate into neurons, we generated a scatter plot of GFAP intensity versus NeuN intensity of all GFP-expressing cells. GFP-expressing cells in the control group (Fig. 3c, blue dots) show low NeuN and high GFAP intensity forming a distinct cluster in the left upper quadrant (G+/N−) (Fig. 3c). GFP-expressing cells in the Neurog2 group (Fig. 3c, pink dots) switched to the right direction in general, indicating that the cells express stronger NeuN signal than those in the control group. The cells in the right lower quadrant (G−/N+) might be completely transdifferentiated into neurons, whereas the cells in the right upper quadrant (G+/N+) are in the transition from astrocytes to neurons (Fig. 3c). In the Neurog2 group, GFAP-negative (G−) cells occupied almost half of the GFP-expressing cells. Contrary to the Neurog2 group, almost all the GFP-expressing cells in the control group were GFAP-positive (G+) (Fig. 3d). Although GFP-expressing cells exhibited strong astrocyte properties with high GFAP intensity and low NeuN intensity in the control group (Fig. 3e), the cells in the Neurog2 group exhibited much more neuron-like properties with increased NeuN and decreased GFAP intensities, reaching a similar level of intensity (Fig. 3f). In other words, GFP-expressing cells in the Neurog2 group expressed higher NeuN (Fig. 3g) and lower GFAP intensity than those of the control group (Fig. 3h). Taken together, the reactive astrocytes transdifferentiated into more neuron-like cells in the brain of non-human primates by sole expression of *Neurog2*.

### Transdifferentiation of scar-forming reactive astrocytes into functional neurons in the SCI mouse model

**Fig. 4:**
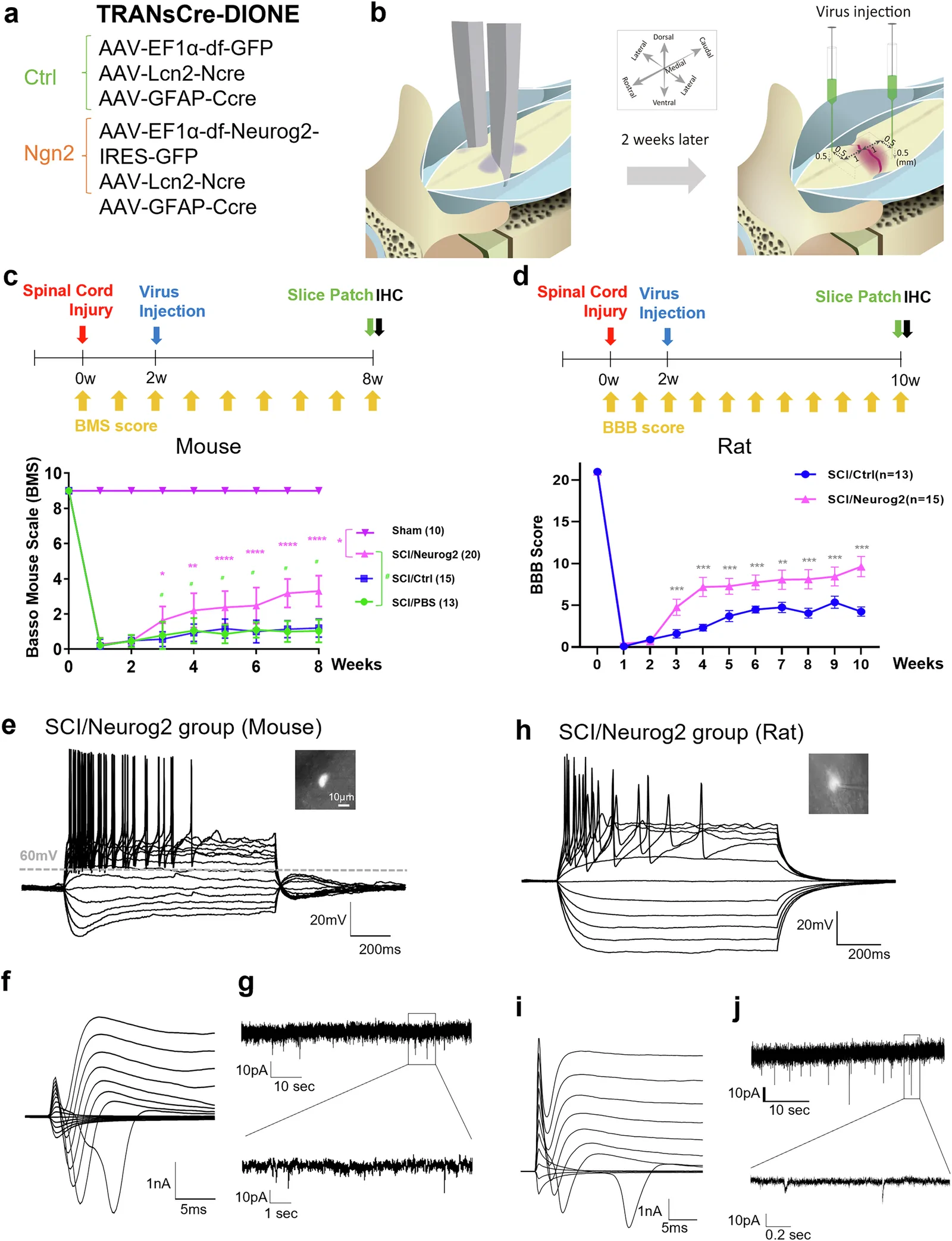
Transdifferentiation of scar-forming reactive astrocytes into functional neurons in the spinal cord injury mouse model. **a** A mixture of three viruses (TRANsCre-DIONE, 1 μl of mixture) was injected into the spinal cord. Basso mouse scale (BMS) was measured in every week from the first week of the injury to the last week. Spinal cord injury (SCI) mice were sacrificed 8 weeks after injury (PI 6 weeks) for immunohistochemistry (IHC) and ex vivo patch-clamp recording. **b** Schematic diagram of the protocol for setting the SCI model and virus injection in the spinal cord. The SCI mouse model was set by forceps compressing protocol (compression for 3 s, at Thoracic 10). A mixture of three viruses (TRANsCre-DIONE, 1 μl of mixture, upper and lower of 1 mm of the injury site) was injected into the spinal cord 2 weeks after injury. **c** BMS scores of sham, SCI/Neurog2, SCI/Ctrl, and SCI/phosphate-buffered saline (PBS) groups. BMS scores were measured every week following the functional recovery of the SCI mouse model (magenta, sham group; pink, SCI/Neurog2 group; blue, SCI/Ctrl group; green, SCI/PBS group). Dunnett’s multiple comparison test, with individual variances computed for each comparison (^#^*P* < 0.05). *N* = 10 (sham group), 20 (SCI/Neurog2 group), 15 (SCI/Ctrl group), and 13 (SCI/PBS group). **d** Basso, Beattie, and Bresnahan (BBB) scores of SCI/Neurog2 and SCI/Ctrl groups. BBB score was measured every week following the functional recovery of the SCI rat model (pink, SCI/Neurog2 group; blue, SCI/Ctrl group). Dunnett’s multiple comparison test, with individual variances computed for each comparison (^#^*P* < 0.05). *N* = 15 (SCI/Neurog2 group), 13 (SCI/Ctrl group). **e**, **h** Action potential firing was in GFP-expressing cell (inset, scale bar, 10 μm) in the SCI/Neurog2 group. Scale bar, 20 mV, 200 ms. **f**, **i** Large sodium channel current was shown in voltage-clamp recording from GFP-expressing cell in the Neurog2 group. Scale bar, 1 nA, 5 ms. **g**, **j** Spontaneous excitatory postsynaptic currents from GFP-expressing cell in the Neurog2 group. Scale bar, 10 pA, 10 s (upper); 10 pA, 1 s (enlarged trace).

To investigate whether the transdifferentiated neurons from reactive astrocytes can ameliorate the motor impairment caused by SCI, we generated the SCI mouse and rat model with forceps compressing of spinal cord at thoracic vertebra (T10) (Fig. 4b) and injected TRANsCre-DIONE (AAV-EF1α-DIO-Neurog2-IRES-EGFP with AAV-Lcn2-NCre and AAV-GFAP-CCre) into 1 mm above and below the injury site 2 weeks after injury (Fig. 4a, b). Starting from the time of injury, every week we measured the BMS (score of 0–9) for mouse and Basso, Beattie, and Bresnahan locomotor rating scale (BBB, score of 0–21) for the rat which represents locomotion defects during recovery (Fig. 4c, d and Supplementary Video 1). The impaired movement of SCI mice was significantly ameliorated to an average BMS score of 3.88 at 8 weeks in the SCI/Neurog2 group, whereas the mice in SCI/Ctrl and SCI/PBS groups scored below an average of 1 (Fig. 4c). The impaired movement of SCI rats was significantly ameliorated to an average BBB score of 9.63 at 10 weeks in the SCI/Neurog2 group, whereas the rats in the SCI/Ctrl group remained significantly lower (Fig. 4d). These results indicate that TRANsCre-DIONE can alleviate the motor impairment of SCI mouse and rat model.

Furthermore, to investigate whether the transdifferentiated cells display functional properties of neurons, GFP-expressing cell (inset, Fig. 4e, h) in the Neurog2 group was recorded by whole-cell patch clamp. GFP-expressing cells showed robust action potential firing (Fig. 4e, h), large sodium currents (Fig. 4f, i), and numerous spontaneous EPSCs (Fig. 4g, j). Taken together, TRANsCre-DIONE causes an improvement of motor behavior and transdifferentiation into functional neurons in SCI.

Additionally, to verify whether the transdifferentiated neurons are functionally integrated into the existing neural circuits and contribute to behavioral recovery, we employed a chemogenetic approach using DREADD (Designer Receptors Exclusively Activated by Designer Drugs). In the mouse SCI model (Supplementary Fig. 5b), we expressed the inhibitory DREADD, hM4Di, specifically in the transdifferentiated neurons using the TRANsCre-DIONE system. Upon administration of clozapine *N*-oxide, we observed a significant reversal of the motor functional recovery previously gained in the SCI/Neurog2 group. Electrophysiological recordings or behavioral assays confirmed that the temporary silencing of these newly formed neurons led to a recurrence of motor deficits, whereas the vehicle-treated group maintained their recovered state. These results provide strong evidence that the transdifferentiated neurons are not merely present but are functionally active and essential for the observed symptomatic alleviation.

### Transcriptomic profiling and safety of neuronal reprogramming

**Fig. 5:**
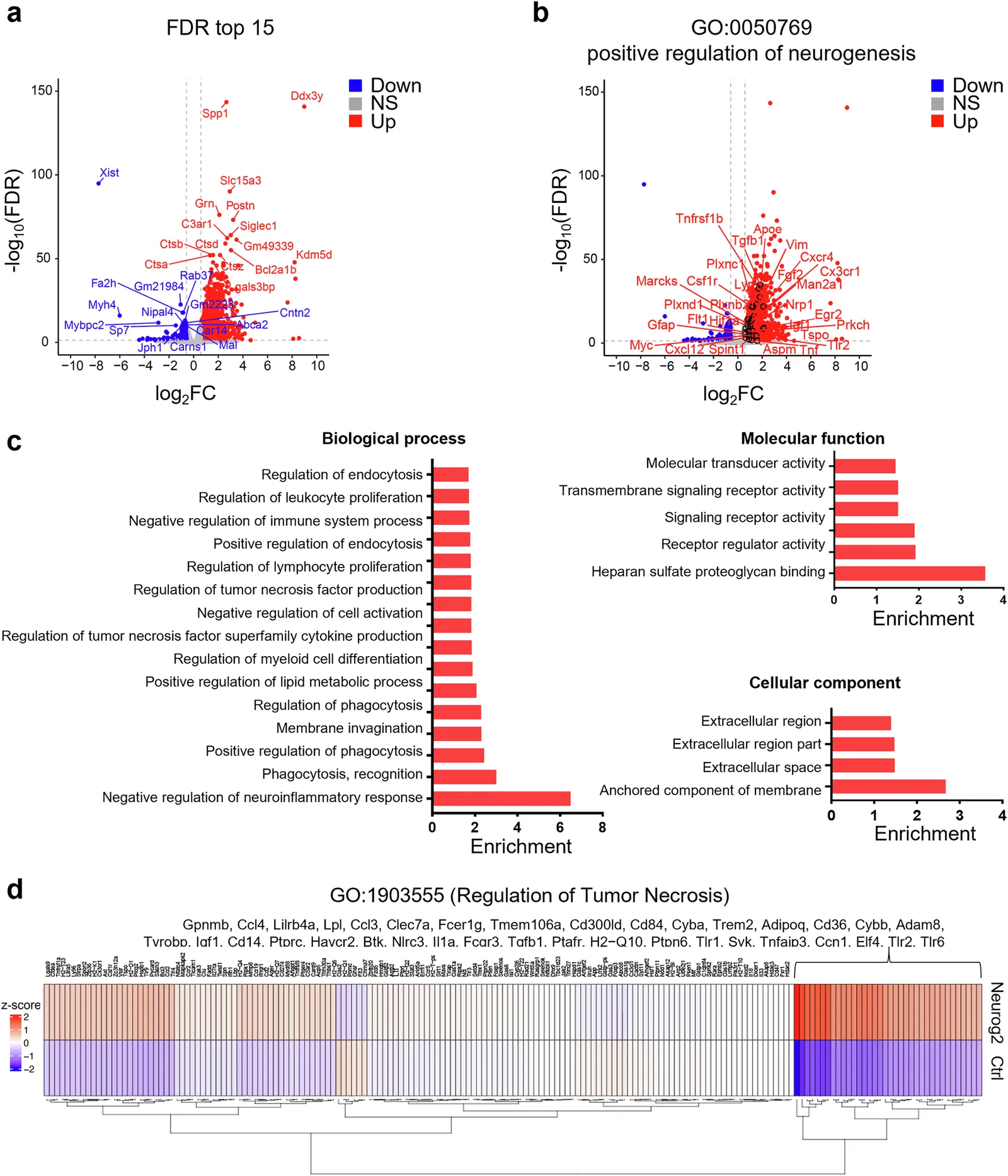
Transcriptomic profiling of Neurog2-mediated neuronal reprogramming and assessment of long-term safety. **a** Volcano plot showing differentially expressed genes between the spinal cord injury (SCI)/control (Ctrl) and SCI/Neurog2 groups (Neurog2 versus Ctrl). Red and blue dots represent significantly upregulated and downregulated genes, respectively. Genes were classified as significantly upregulated (log_2_ fold change > 0.585, false discovery rate (FDR) < 0.05) or downregulated (log_2_ fold change < −0.585, FDR < 0.05). The top 15 upregulated and downregulated genes ranked by FDR are labeled. **b** Volcano plot highlighting neurogenesis-related genes. Genes associated with the Gene Ontology (GO) term for positive regulation of neurogenesis (GO: 0050769) are highlighted within the global transcriptomic distribution. **c** GO analysis of pathways associated with downregulated (blue) and upregulated (red) genes. Bar graphs represent the enrichment of terms related to SCI/Neurog2 compared to SCI/Ctrl group. The *x*-axis represents the enrichment scores for specific biological processes, molecular functions, and cellular components. **d** Heatmap of genes detected in RNA-seq result belongs to the GO: 1903555, Regulation of tumor necrosis. The transcriptomic profile shows a clear shift in the Neurog2-treated group, exhibiting a distinct signature compared with the SCI/Ctrl group. The expression levels are represented by *Z*-scores.

To elucidate the molecular mechanism of *Neurog2*-mediated transdifferentiation and evaluate its gene profiling change and safety at the transcriptomic level, we performed bulk RNA-seq on the SCI lesion site (Fig. 5). Volcano plot revealed consistent transcriptomic differences between the SCI/Neurog2 and SCI/Ctrl groups (Fig. 5a), identifying differentially expressed genes (DEGs) associated with the reprogramming process (Fig. 5b, c). Notably, Gene Ontology (GO) analysis of these DEGs revealed a significant enrichment of genes associated with the positive regulation of neurogenesis (GO:0050769), confirming the activation of neurogenic programs in the SCI/Neurog2 group (Fig. 5b). Further functional enrichment analysis (Fig. 5c) demonstrated a significant shift in biological processes; upregulated DEGs were notably associated with the negative regulation of neuroinflammatory response and phagocytosis, suggesting a favorable microenvironment for neuronal integration (Fig. 5c).

Crucially, from a safety perspective, the transcriptomic profile showed a shift away from signatures associated with pluripotency or uncontrolled proliferation, which are primary concerns in cell-based therapies regarding teratoma formation. This transition is further supported by heatmap analysis of genes related to the regulation of tumor necrosis (GO:1903555), which showed a consistent and distinct expression pattern in the Neurog2 group compared with the control group (Fig. 5d). This directed reprogramming, combined with the lack of Ki67/BrdU incorporation in GFP+/NeuN+ cells (Fig. 8), reinforces the conclusion that TRANsCre-DIONE is a safe and robust strategy for in vivo neuronal replacement without tumorigenic potential. In summary, our transcriptomic analyses suggest that Neurog2-mediated reprogramming is associated with a shift toward a neuronal identity with controlled proliferative activity, supporting its potential for in vivo neuronal replacement.

### Tissue recovery in SCI via transdifferentiation of scar-forming reactive astrocytes into neurons

**Fig. 6:**
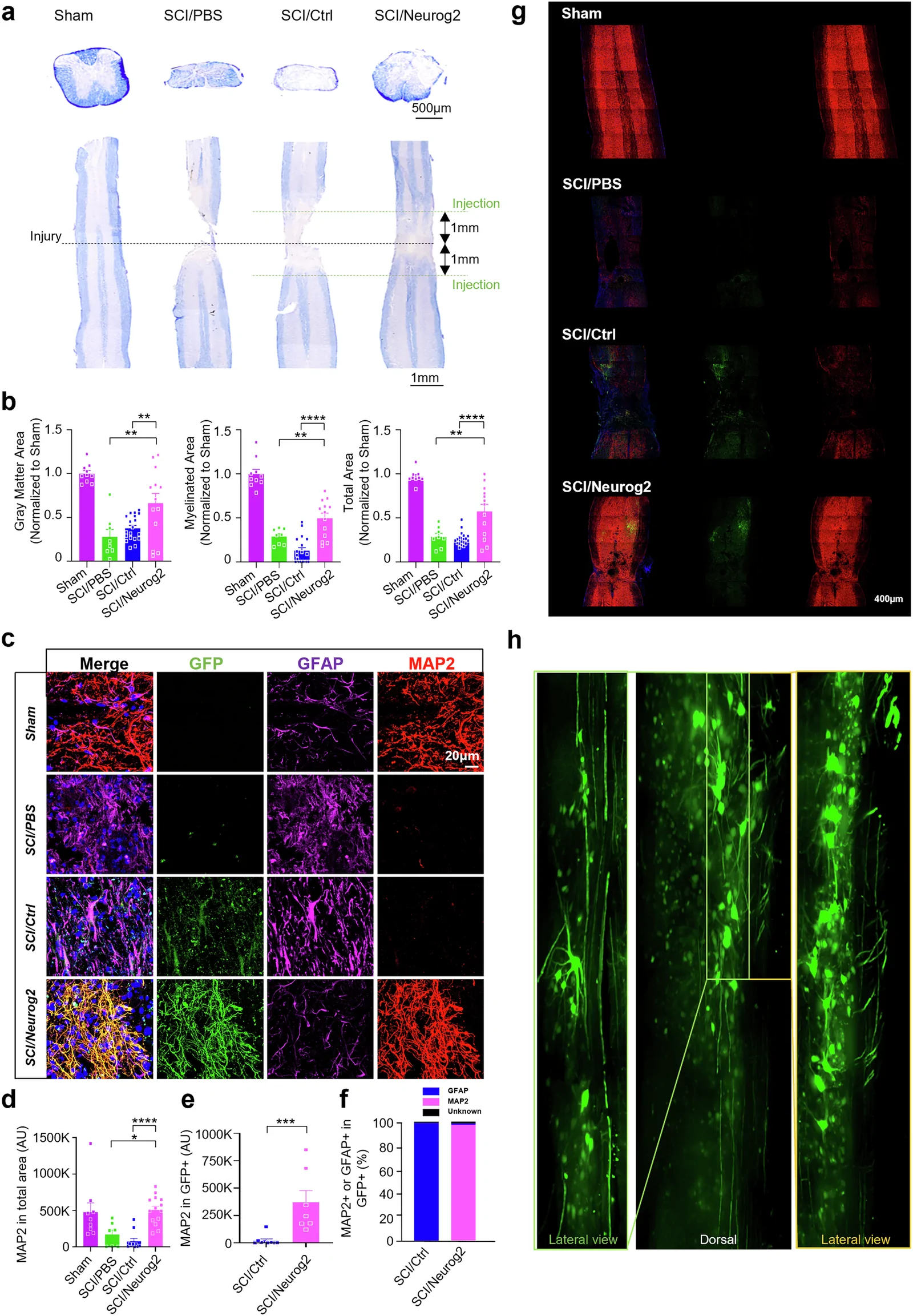
Tissue recovery in SCI via transdifferentiation of scar-forming reactive astrocytes into neurons. **a** Eriochrome Cyanine (EC) for myelin staining in cross and longitudinal sections in sham, spinal cord injury (SCI)/phosphate-buffered saline (PBS), SCI/control (Ctrl), and SCI/Neurog2 groups (black dot line; injury site, green dot line; injection site). Scale bar, 500 μm (cross-section), 1 mm (longitudinal section). **b** Scattered bar graph showing the area of gray matter (left), myelinated area (middle), and total area (right) in sham (magenta), SCI/PBS (green), SCI/Ctrl (blue), and SCI/Neurog2 (pink) groups (each value was normalized by the sham group). Unpaired non-parametric one-way analysis of variance (ANOVA) with Kruskal–Wallis test. *n* = 10 (sham group), 8 (SCI/PBS group), 19 (SCI/Ctrl group), 13 (SCI/Neurog2 group). **c** Colocalization of GFAP (magenta) and MAP2 (red) with GFP fluorescence (green) in sham, SCI/PBS, SCI/Ctrl, and SCI/Neurog2 groups. Scale bar, 20 μm. **d** Scattered bar graph showing comparison of MAP2 intensity in the total area. Multiple comparison for unpaired non-parametric one-way ANOVA with Kruskal–Wallis test. *n* = 9 (sham group), 7 (SCI/PBS group), 11 (SCI/Ctrl group), and 14 (SCI/Neurog2 group). **e** Scattered bar graph showing comparison of MAP2 intensity in GFP-expressing cells in SCI/Ctrl and SCI/Neurog2 groups. Unpaired non-parametric *t*-test with Mann–Whitney test. *n* = 11 (SCI/Ctrl group), 14 (SCI/Neurog2 group). **f** Stacked bar graph showing the proportion of GFAP (blue)-positive or MAP2 (pink)-positive cells in GFP-expressing cells (undefined: black). **g** MAP2 staining of longitudinal sections of sham, SCI/PBS, SCI/Ctrl, and SCI/Neurog2 groups. GFP (green), red (MAP2). Scale bar, 400 μm. **h** GFP-expressing transdifferentiated neurons (signals were not amplified). Light-sheet 3D image. SCI/Neurog2 group.

To test whether overexpression of *Neurog2* in reactive astrocytes causes tissue recovery, we performed Eriochrome Cyanine (EC) staining with the spinal cord tissues to measure the myelinated, gray matter, and total areas (Fig. 6a). Although the tissues were severely shrunken and damaged in the SCI/PBS and SCI/Ctrl groups, tissues in the Neurog2 group were recovered almost to the sham group (Fig. 6a). The gray matter, myelinated, and total areas were decreased in SCI/PBS (27.95%, 28.77%, 28.39%) and SCI/Ctrl (37.74%, 12.69%, 26.01%) groups compared with the sham group, and the areas were significantly increased in the SCI/Neurog2 group (66.37%, 49.51%, 57.45%) (Fig. 6b).

To explore whether the damaged axon and dendrites were recovered in an extensive field, we observed the longitudinal view of MAP2 and 3D imaged cleared spinal cord (SCI/Neurog2 group) by light-sheet microscopy (Fig. 6g,h and Supplementary Video 2). MAP2 signal disappeared in SCI/PBS and SCI/Ctrl groups and significantly restored in the SCI/Neurog2 group (Fig. 6c,d,g). MAP2 signals in GFP-expressing cells were also significantly increased in SCI/Neurog2 group compared with SCI/Ctrl group (Fig. 6c, e). About 97.32% of the GFP-expressing cells were MAP2-positive in the SCI/Neurog2 group, whereas 98.88% of the GFP-expressing cells were GFAP-positive in the SCI/Ctrl group (Fig. 6f). To generate 3D image showing regenerated neurons (GFP) across the injury in recovered spinal cord (SCI/Neurog2), the spinal cords were cleared by SHIELD passive clearing protocol (Life Canvas Technologies)^47^, and we took images from transparent tissues by light-sheet microscope (Lightsheet 7, ZEISS) (Fig. 6h and Supplementary Video 2). These results indicate that the most of the GFP-expressing cells were transdifferentiated into MAP2+ neurons. Furthermore, the myelinated axon and the damaged tissue structure might be restored via TRANsCre-DIONE.

### Transdifferentiation of reactive astrocytes into motor neurons in SCI

Isl1 is necessary for differentiating most of the subtypes of motor neurons such as VAChT+, ChAT+, CHT+ neurons, and Isl1-expressing cholinergic motor neurons are major cell types in the spinal cord^48^. To examine whether overexpression of *Neurog2* induces transdifferentiation of reactive astrocytes into specific neurons, we immunostained the spinal cord tissues with antibodies against both Isl1 and ChAT, the marker for motor neuron^43^ with NeuN, the mature neuronal marker.

**Fig. 7:**
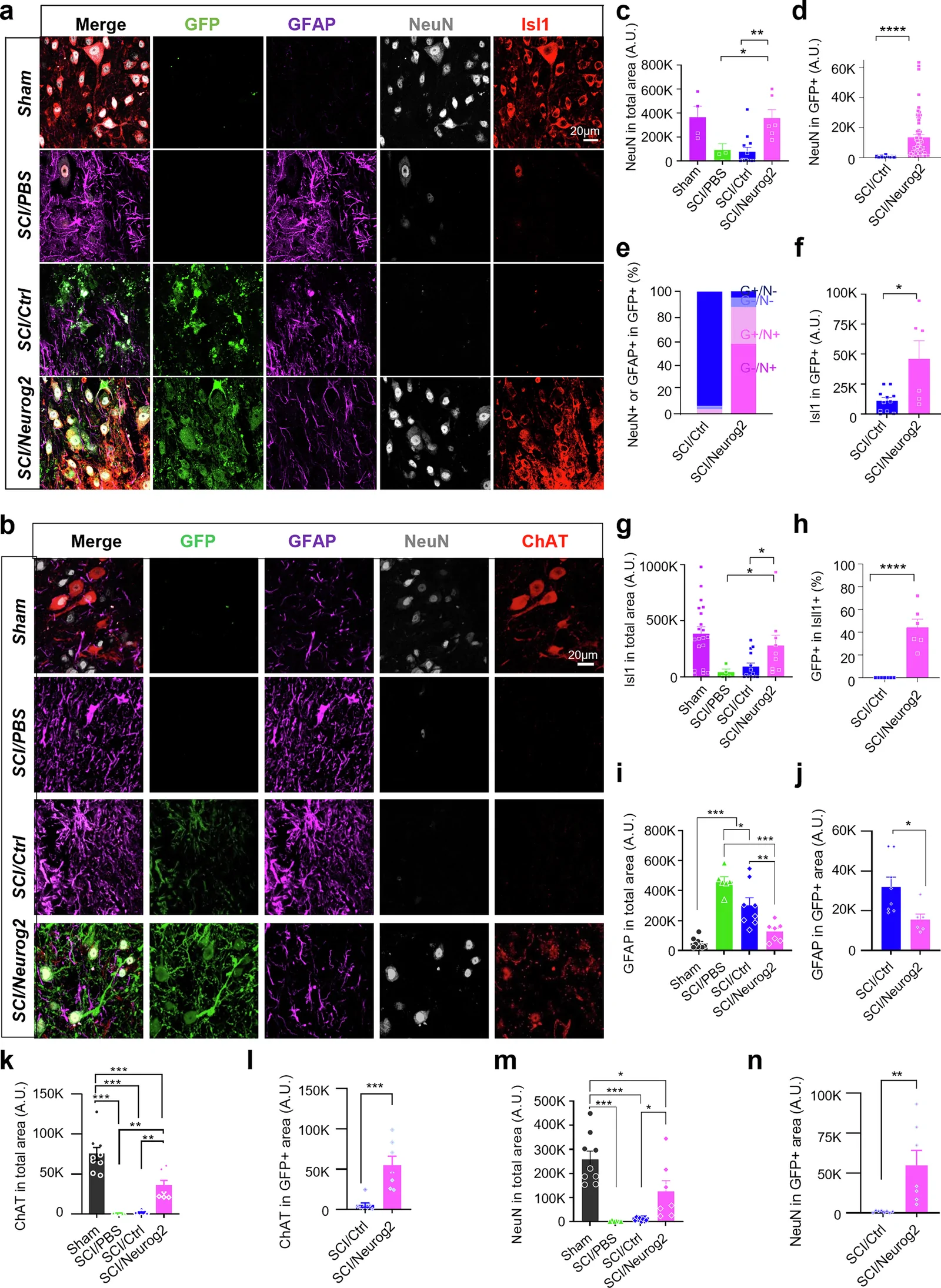
Transdifferentiation of reactive astrocytes into motor neurons in SCI. **a** Colocalization of GFAP (magenta), NeuN (gray), and Isl1 (red) with GFP fluorescence (green) in sham, spinal cord injury (SCI)/phosphate-buffered saline (PBS), SCI/control (Ctrl) and SCI/Neurog2 groups. Scale bar, 20 μm. **b** Colocalization of GFAP (magenta), NeuN (gray), and ChAT (red) with GFP fluorescence (green) in sham, SCI/PBS, SCI/Ctrl, and SCI/Neurog2 groups. Scale bar, 20 μm. **c** Scattered bar graph showing comparison of NeuN intensity in the total area. Multiple comparison for unpaired non-parametric one-way analysis of variance (ANOVA) with Kruskal–Wallis test. *n* = 4 (sham group), 4 (SCI/PBS group), 13 (SCI/Ctrl group), 6 (SCI/Neurog2 group). **d** Scattered bar graph showing comparison of NeuN intensity in GFP-expressing cells in SCI/Ctrl and SCI/Neurog2 groups. Unpaired non-parametric *t* test with Mann–Whitney test. *n* = 66 (SCI/Ctrl group), 65 (SCI/Neurog2 group). **e** Stacked bar graph showing the proportion of GFAP and NeuN positive or negative cells in GFP-expressing cells. G+/N− (blue, quadrant 2 of Supplementary Fig. 5d), G−/N− (light blue, quadrant 3 of Supplementary Fig. 5d), G+/N+ (light pink, quadrant 1 of Supplementary Fig. 5d), and G−/N+ (pink, quadrant 4 of Supplementary Fig. 5d). *n* = 102 in SCI/Ctrl group and *n* = 77 in SCI/Neurog2 group. **f** Scattered bar graph showing comparison of Isl1 intensity in GFP-expressing cells in SCI/Ctrl and SCI/Neurog2 groups. Unpaired non-parametric *t* test with Mann–Whitney test. *n* = 11 (SCI/Ctrl group) and 6 (SCI/Neurog2 group). **g** Scattered bar graph showing comparison of Isl1 intensity in the total area. Multiple comparison for unpaired non-parametric one-way analysis of variance (ANOVA) with Kruskal–Wallis test. *n* = 20 (sham group), 4 (SCI/PBS group), 14 (SCI/Ctrl group), 9 (SCI/Neurog2 group). **h** Scattered bar graph showing comparison of GFP-expressing cells in Isl1-positive cells in SCI/Ctrl and SCI/Neurog2 groups. Unpaired non-parametric *t*-test with Mann–Whitney test. *n* = 7 (SCI/Ctrl group) and 6 (SCI/Neurog2 group). **i** Scattered bar graph showing comparison of GFAP intensity in the total area. Multiple comparison for unpaired non-parametric one-way ANOVA with Kruskal–Wallis test. *n* = 10 (sham group), 8 (SCI/PBS group), 18 (SCI/Ctrl group), 24 (SCI/Neurog2 group). **j** Scattered bar graph showing comparison of GFAP intensity in GFP-expressing cells in SCI/Ctrl and SCI/Neurog2 groups. Unpaired non-parametric *t*-test with Mann–Whitney test. *n* = 8 (SCI/Ctrl group) and 6 (SCI/Neurog2 group). **k** Scattered bar graph showing comparison of ChAT intensity in the total area. Multiple comparison for unpaired non-parametric one-way ANOVA with Kruskal–Wallis test. *n* = 4 (sham group), 4 (SCI/PBS group), 13 (SCI/Ctrl group), and 6 (SCI/Neurog2 group). **l** Scattered bar graph showing comparison of ChAT intensity in GFP-expressing cells in SCI/Ctrl and SCI/Neurog2 groups. Unpaired non-parametric *t*-test with Mann–Whitney test. *n* = 66 (SCI/Ctrl group) and 65 (SCI/Neurog2 group). **m** Scattered bar graph showing comparison of NeuN intensity in the total area. Multiple comparison for unpaired non-parametric one-way ANOVA with Kruskal–Wallis test. *n* = 9 (sham group), 6 (SCI/PBS group), 8 (SCI/Ctrl group), 7 (SCI/Neurog2 group). **n** Scattered bar graph showing comparison of NeuN intensity in GFP-expressing cells in SCI/Ctrl and SCI/Neurog2 groups. Unpaired non-parametric *t* test with Mann–Whitney test. *n* = 8 (SCI/Ctrl group) and 7 (SCI/Neurog2 group).

NeuN signal was also disappeared in SCI/PBS and SCI/Ctrl groups and significantly recovered in the SCI/Neurog2 group (Fig. 7a, c). NeuN signal in GFP-expressing cells was also significantly increased in the SCI/Neurog2 group compared with the SCI/Ctrl group (Fig. 7a, d). To determine the transdifferentiation efficiency and specificity, we performed detailed analysis of GFP-expressing cells by plotting NeuN versus GFAP (Supplementary Fig. 5c, d). We found that 87% (67 out of 77 cells) of GFP-expressing cells were NeuN-positive in the SCI/Neurog2 group, whereas only 3.92% (4 out of 102 cells) were NeuN-positive in the SCI/Ctrl group (Fig. 7e). The Isl1 signal was disappeared in SCI/PBS and SCI/Ctrl groups and significantly increased in the SCI/Neurog2 group (Fig. 7a, g). The Isl1 signal in GFP-expressing cells was also significantly increased in the SCI/Neurog2 group compared with the SCI/Ctrl group (Fig. 7a, f). The percentage of Isl1+/GFP+ motor neurons in total Isl1+ motor neurons was significantly increased in the SCI/Neurog2 group, whereas all the Isl1+ motor neurons were GFP− in the SCI/Ctrl group (Fig. 7a,h). Similarly, the ChAT or NeuN signal was also disappeared in SCI/PBS and SCI/Ctrl groups and significantly increased in the SCI/Neurog2 group (Fig. 7b, k, m). The ChAT+ or NeuN+ cells in GFP+ area were also significantly increased in the SCI/Neurog2 group compared with the SCI/Ctrl group (Fig. 7b, l, n). These results indicate that reactive astrocytes transdifferentiated into Isl1+ or ChAT+ motor neurons in the injured spinal cord by overexpression of *Neurog2*. Moreover, to explore whether overexpression of *Neurog2* in reactive astrocytes affects the reactivity of astrocytes, we immunostained the tissues with the antibody against GFAP. Although the GFAP signal was increased in SCI/PBS and SCI/Ctrl groups, GFAP was significantly decreased in the SCI/Neurog2 group (Fig. 7a, i, j). Taken together, TRANsCre-DIONE induces transdifferentiation of reactive astrocytes into motor neurons and attenuates the reactivity of astrocytes in the injured spinal cord.

### Transdifferentiated neurons mostly originate from non-proliferating cells

**Fig. 8:**
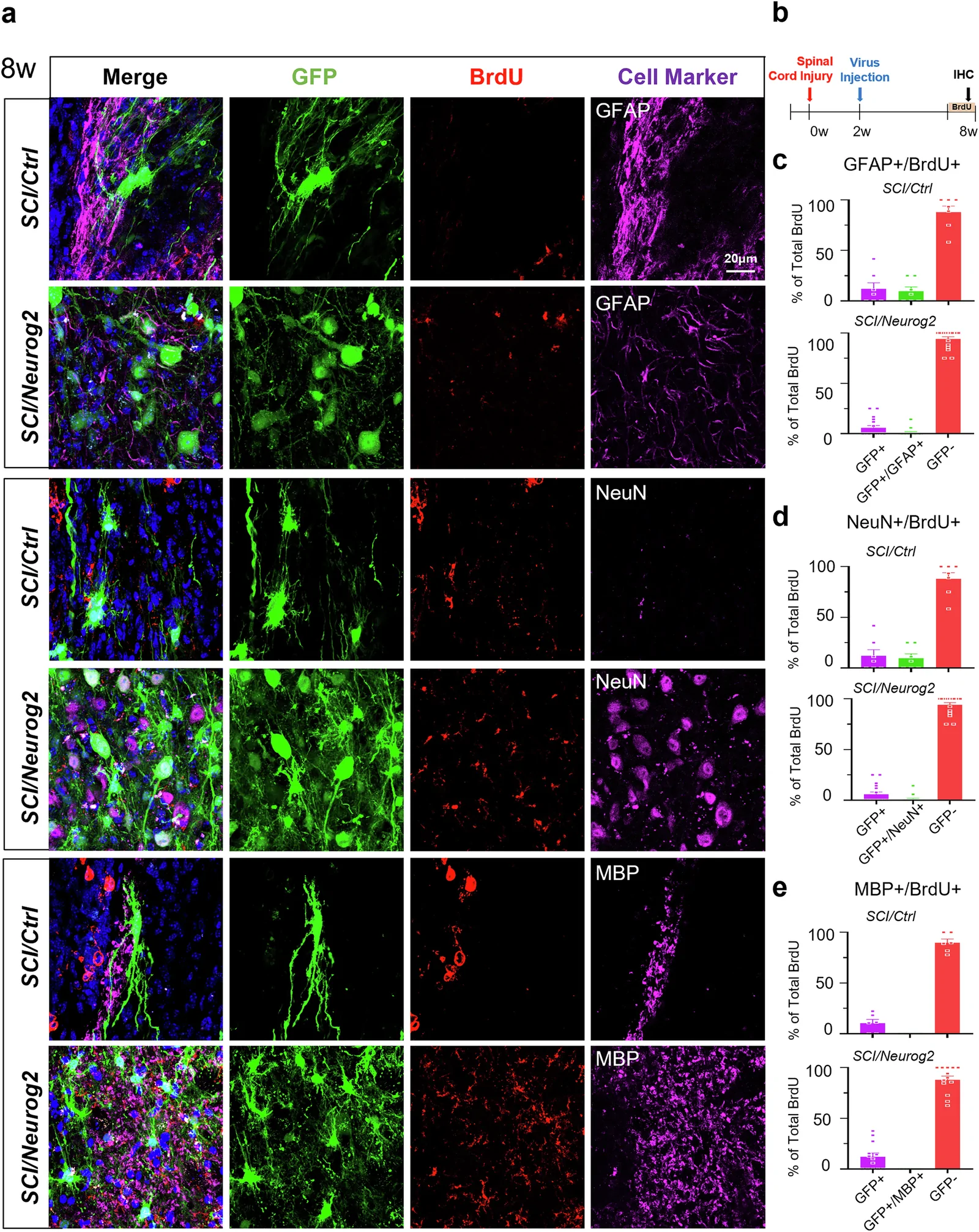
Transdifferentiated neurons mostly originate from non-proliferating cells. **a** Colocalization of BrdU (red, treated for 1 week from 7 weeks after injury) in GFP-expressing cells (green) with cell markers (magenta, GFAP; upper, NeuN; middle, MBP, lower) in spinal cord injury (SCI)/control (Ctrl) and SCI/Neurog2 groups. Scale bar, 20 μm. **b** Timeline of treating BrdU and immunohistochemistry after sacrificing in SCI mice. **c** Scattered bar graph showing comparison of percentage of BrdU-positive cells in GFP+, GFAP+/GFP+, and GFP− cells in SCI/Ctrl and SCI/Neurog2 groups. *n* = 7 (SCI/Ctrl group), 17 (SCI/Neurog2 group). **d** Scattered bar graph showing comparison of percentage of BrdU-positive cells in GFP+, NeuN+/GFP+, and GFP− cells in SCI/Ctrl and SCI/Neurog2 groups. *n* = 7 (SCI/Ctrl group), 17 (SCI/Neurog2 group). **e** Scattered bar graph showing comparison of percentage of BrdU-positive cells in GFP+, MBP+/GFP+, and GFP− cells in SCI/Ctrl and SCI/Neurog2 groups. *n* = 7 (SCI/Ctrl group) and 17 (SCI/Neurog2 group).

To investigate whether the transdifferentiated neurons are derived from proliferating cells, we performed labeling with BrdU which is incorporated into DNA during cell proliferation and neurogenesis^49^ or Ki67 which is a protein that is expressed in actively dividing cells. BrdU was administered once a day for 1 week to the SCI mice before they were sacrificed at the time of 3 (Supplementary Fig. 6) and 8 weeks (Fig. 8a, b). For measuring proportion of BrdU+ or Ki67+ cells in diverse GFP-expressing cell types, we double-stained the tissues with antibodies against GFAP or NeuN or MBP (myelin basic protein), the cell markers of astrocyte, neuron, and oligodendrocyte, respectively (Fig. 8a and Supplementary Figs. 6a, 7a, and 8a). In the SCI/Ctrl group, only 12.06% of the GFP+ cells and 9.60% of the GFP+/GFAP+ cells were labeled with BrdU, whereas most of the GFP− cells were labeled with BrdU (87.90%, Fig. 8c). Similarly, in the SCI/Neurog2 group, only 5.90% of the GFP+ cells and 1.18% of the GFP+/GFAP+ cells were labeled with BrdU, while almost all of the GFP− cells were labeled with BrdU (94.09%, Fig. 8c). These results indicate that most of the GFP+ cells are not proliferating in the late stage (Fig. 8), as well as in the early stage of transdifferentiation (Supplementary Fig. 6). Double labeling with BrdU and NeuN resulted in BrdU labeling of only 10.36% of the GFP+ cells and 0.39% of the GFP+/NeuN+ cells in the SCI/Ctrl group. Similarly, BrdU labeled only 9.82% of the GFP+ cells and 9.62% of the GFP+/NeuN+ cells in the SCI/Neurog2 group (Fig. 8d). By contrast, most of the GFP− cells in the SCI/Ctrl group (89%) and in the SCI/Neurog2 group (85%) were labeled with BrdU (Fig. 8d). These results indicate that most of the transdifferentiated GFP+/NeuN+ cells are not proliferating in the late stage (Fig. 8a, d), as well as in the early stage of the transdifferentiation (Supplementary Fig. 6d). There were no GFP+/MBP+ cells in both SCI/Ctrl and SCI/Neurog2 groups, indicating that oligodendrocytes were not transdifferentiating into neurons (Fig. 8e and Supplementary Fig. 6e). Despite staining more cells than BrdU because Ki67 is expressed in the nucleus during all active phases of the cell cycle, Ki67 labeling with cell markers shows a similar tendency to the BrdU result (Supplementary Figs. 7 and 8). Taken together, these results demonstrate that GFP+ transdifferentiated neurons mostly originate from BrdU-negative non-proliferating cells in the early stage as well as in the late stage of transdifferentiation.

To investigate whether the reduction of reactive astrocytes influences the proliferation of surrounding cells (BrdU+/GFP−) near the transdifferentiated neurons, we assessed the proportion of BrdU+ cells in each cell type (GFAP+, NeuN+, MBP+) among the surrounding GFP− cells (Table 1). We found that the proportion of proliferating astrocytes near the transdifferentiated cells (GFP−/BrdU+/GFAP+) was decreased from 9.62% (SCI/Ctrl) to 4.55% (SCI/Neurog2) in the early stage (3 weeks) and from 12.50% (SCI/Ctrl) to 10.20% (SCI/Neurog2) in the late stage (8 weeks) (Table 1). The amount of BrdU or Ki67 at 3 weeks and the BrdU at 8 weeks were also calculated, all groups show similar tendency with Fig. 8. By contrast, the proportion of GFP−/BrdU+/NeuN+ cells was increased from 0.00% (SCI/Ctrl) to 4.49% (SCI/Neurog2) in the early stage (3 weeks) and from 1.93% to 9.80% in the late stage of transdifferentiation (8 weeks) (Table 1). Consistently, the proportion of GFP−/BrdU+/MBP+ cells was increased from 9.13% (SCI/Ctrl) to 12.03% (SCI/Neurog2) in the early stage (3 weeks) and from 1.33% to 11.94% in the late stage of transdifferentiation (8 weeks) (Table 1). Taken together, these results implicate that the regeneration of neighboring neurons (GFP−/BrdU+/NeuN+) and oligodendrocytes (GFP−/BrdU+/MBP+) was enhanced when the astrocytic reactivity was decreased by TRANsCre-DIONE in the SCI/Neurog2 group in both early and late stages.

**Table 1:** Proportion of non-transdifferentiated (GFP−) proliferative (BrdU+) astrocytes (GFAP+), neurons (NeuN+), and oligodendrocytes (MBP+).

		GFAP		NeuN		MBP	
		SCI/Ctrl	SCI/Neurog2	SCI/Ctrl	SCI/Neurog2	SCI/Ctrl	SCI/Neurog2
3 weeks	Total BrdU+	52	22	138	156	230	158
	GFP−/BrdU+/Marker+	5	1	0	7	21	19
		9.62%	4.55%	0.00%	4.49%	9.13%	12.03%
8 weeks	Total BrdU+	64	98	207	204	75	134
	GFP−/BrdU+/Marker+	8	10	4	20	1	16
		12.50%	10.20%	1.93%	9.80%	1.33%	11.94%

## Discussion

In this study, we have developed and characterized a novel molecular tool set, TRANsCre-DIONE, which achieved high efficiency (62% in mouse striatum and 87% in SCI) in replacing dead neurons under severe injury conditions (Supplementary Fig. 9). The key features of this system are as follows: (1) reactive astrocytes are converted into neurons through overexpression of a single transcription factor, *Neurog2*; (2) reactive astrocytes are preferentially targeted using a split-Cre system driven by two promoters (*GFAP* with *Lcn2* in mouse and *GFAP* with *iNOS* in monkey); (3) TRANsCre-DIONE reduces astrogliosis, promotes neuronal replacement, and is associated with functional improvement in SCI; and (4) the induced neurons exhibit region-specific identities, including GABA+ medium spiny neurons in the striatum and Isl1+ or ChAT+ motor neuron-like cells in the spinal cord. TRANsCre-DIONE shows potential for translational application, as the combination of GFAP and iNOS was effective in the striatum of non-human primate. TRANsCre-DIONE not only induced direct reprogramming of the infected cells but also caused an indirect enhancement of regeneration in neighboring cells, possibly due to removing toxic reactive astrocytes.

In our previous studies, we defined “reactive” astrocytes as astrocytes that respond to damaging stimuli — such as traumatic brain injury or toxic protein aggregates — by activating the MAO-B-dependent GABA synthetic pathway as a consequence of the degradation of toxic materials^18,22^. These reactive astrocytes are known to be detrimental to neighboring neurons and can be easily distinguished from “active” astrocytes, which are known to be beneficial, are present in conditions of enriched environment, and show an enhanced expression of proBDNF with no GABA^18,22^. Although the active and reactive astrocytes can be easily distinguished by the two markers, proBDNF and GABA, it is not very easy to distinguish them by the morphology, because both the active and reactive astrocytes show hypertrophy, which is simply an increased level of GFAP expression. In other words, it is very difficult to distinguish between beneficial active astrocytes and detrimental reactive astrocytes using GFAP alone. By utilizing the reactive-astrocyte-specific inducible genes such as *Lcn2* and *iNOS* for promoters of split-Cre in combination with *GFAP* promoter, our split-Cre system was designed to improve the selective targeting of reactive astrocyte populations. Although Lcn2 and iNOS are not exclusively expressed in astrocytes under pathological conditions, our colocalization analyses indicated minimal overlap with non-astrocytic populations, suggesting that the combinatorial strategy enhances targeting specificity (Supplementary Fig. 1). Nevertheless, it is important to note that this approach may preferentially target specific subtypes of reactive astrocytes, particularly inflammation-associated populations, and may not capture the full heterogeneity of reactive astrocytes. We have observed that TRANsCre-DIONE shows a high degree of specificity for reactive astrocytes, targeting more than 90.21% of reactive astrocytes in the injured mouse striatum (Fig. 2c), 93.13% in the injured monkey striatum (Fig. 3c), and 98.48% in SCI (Fig. 6f). However, rather than indicating absolute specificity, these results should be interpreted as reflecting preferential targeting within the defined experimental conditions. Notably, the combination of GFAP and iNOS promoters showed particularly high specificity in non-human primates, supporting the potential translational applicability of this strategy.

The specificity toward reactive astrocytes or neurons can be estimated by calculating the proportion of GFAP+/GFP+ or NeuN+/GFP+ cells in total GFP+ cells in the Ctrl group. In other words, the specificity toward NeuN+ neurons in the Ctrl group represents the leakiness of TRANsCre-DIONE toward resident neurons. In the Ctrl group, we observed that only 1 out of 93 (1.07%) GFP+ cells was NeuN+ in the mouse striatum (Fig. 2c), 1 out of 98 (1.03%) GFP+ cells was NeuN+ in the monkey’s striatum (Fig. 3c), and 4 out of 102 (3.92%) GFP+ cells was NeuN+ in the mouse spinal cord (Supplementary Fig. 5c, d). These results indicate that TRANsCre-DIONE shows minimal leakiness toward resident neurons. This is in contrast to previous GFAP-based systems, which have reported higher baseline neuronal labeling, potentially leading to overestimation of transdifferentiation efficiency. Therefore, the efficiency values reported here are less likely to be confounded by such effects. The high transdifferentiation efficiency of TRANsCre-DIONE can be attributed to the use of DIO system^35^ in combination with the strong *EF1α* promoter^36^. This design enables sustained expression of Neurog2 even as astrocytes transition toward a neuronal identity, overcoming the limitations of GFAP promoter-driven expression. In addition, Cre-dependent activation prevents ectopic expression of Neurog2 in untargeted cells, contributing to the low level of leakiness observed.

Our study reveals that the sole use of the transcription factor, *Neurog2*, is a promising candidate for transdifferentiation of reactive astrocytes into neurons. We found that *Neurog2* alone is capable of transdifferentiating reactive astrocytes — in cortical astrocyte culture, mouse and monkey striatum, and mouse spinal cord — into neurons without a need for additional transcription factors. More importantly, *Neurog2* possesses a chameleon-like property that allows transdifferentiation of reactive astrocytes into various neuronal types depending on the surrounding environment and region of the injury site. It has been shown that *Neurog2* can transdifferentiate cultured astrocytes into glutamatergic neurons^12^. It has been also shown that *Neurog2* can transdifferentiate astrocytes into glutamatergic neurons or GABAergic neurons in vivo in cortex, cerebellum, and spinal cord, although the majority was glutamatergic^41^. However, the transdifferentiation efficiency was less than 10% probably due to the use of *GFAP* promoter alone to drive *Neurog2* expression^41^. We have shown that *Neurog2* under TRANsCre-DIONE can transdifferentiate reactive astrocytes into GABA+ medium spiny neurons at high rate (83.64%) in the mouse striatum (Fig. 2d, e). In SCI, *Neurog2* was capable of transdifferentiating reactive astrocytes into isl1+ cholinergic motor neurons (Fig. 7a, g, h). These results imply that the same transcription factor *Neurog2* can cause a transdifferentiation of the target cells into different types of neurons and that the environmental factors strongly influence the fate of the *Neurog2*-expressing transdifferentiating cells. How is this possible for *Neurog2*? As an upstream master switch gene, *Neurog2* is known to induce sequential expression of other neurogenesis-related downstream transcription factors such as *NeuroD1, NeuroD4, Hes6*, and *MyT1* (ref. ^38^). Thus, *Neurog2* has a potential to induce transdifferentiation into diverse types of neurons by turning on a specific switch depending on the niche of the residential brain regions. Although our study did not directly dissect these pathways, transcriptomic analysis provides initial insights into the molecular changes associated with reprogramming and supports the activation of neuronal gene programs. Future studies will be required to further define the regulatory networks underlying this process. Unlike *Neurog2*, *NeuroD1* appears to transdifferentiate astrocytes into mostly glutamatergic neurons in hippocampus^29,50^ when it is used solely. *NeuroD1* appears to require other transcription factors such as *DLX2* in combination to transdifferentiate into other types of neurons other than glutamatergic neurons^29,45^. In support of this idea, it has been recently demonstrated that a gene silencing of *PTBP1* induces transdifferentiation of Müller glia to retinal ganglion cells in the retina, whereas it induces transdifferentiation of astrocytes into dopaminergic neurons in the striatum without additional factors^51^. Consistently, another group very recently reported the similar chameleon-like property of *PTBP1*-gene-silencing, in which converted neurons showed a region specificity^52^. It will be interesting to test whether *Neurog2* is the downstream transcription factor of *PTBP1*-gene-silencing. Although a direct experimental comparison with other strategies was not the primary focus of this study, the high efficiency and the successful generation of region-specific motor neurons highlight the distinct advantages of Neurog2 for spinal cord repair.

In this study, we have achieved multiple goals using TRANsCre-DIONE: elimination of detrimental reactive astrocytes, replacement of dead neurons, and alleviation of motor symptoms in SCI model. Mechanistically, Neurog2 is known to activate downstream transcriptional networks, including NeuroD family genes and other neurogenic factors, which may contribute to the observed diversity of neuronal phenotypes. Although our study did not directly dissect these pathways, transcriptomic analysis provides initial insight into the molecular changes associated with reprogramming and supports the activation of neuronal gene programs. It has been proposed that the “scar-forming reactive astrocytes” (defined as “severe reactive astrocytes” in our study) in SCI inhibit axon regeneration under the chronic pathological condition, whereas “reactive astrocytes” (defined as “mild reactive astrocytes” in our study) serve protective roles in acute pathological condition^53^. According to this proposed model, the reactive astrocytes can be transformed into normal astrocytes or scar-forming reactive astrocytes by the surrounding environment in the spinal cord^19^. Thus, we focused on the effect of eliminating the detrimental scar-forming reactive astrocytes to provide a niche for axon regeneration. Consistent with the proposed model, we observed both a significant decrease in GFAP signal in the SCI/Neurog2 group, indicating a relief from astrocytic reactivity and a significant increase in MAP2 signal, indicating an enhanced axon regeneration. In addition, TRANsCre-DIONE synergistically increased appearance of the BrdU+ proliferating cells that were GFP− (Table 1), indicating that removal or reprogramming of reactive astrocytes may indirectly promote a pro-regenerative environment. These findings suggest that functional recovery may arise from both neuronal replacement and modulation of the injury niche, rather than from cell replacement alone.

In conclusion, TRANsCre-DIONE enables efficient and selective reprogramming of reactive astrocytes into neurons across multiple injury contexts. Although the absence of overt tumorigenesis and the avoidance of cell transplantation represent important advantages, comprehensive evaluation of long-term safety, off-target effects, and functional integration will be essential for future translational applications. Finally, the feasibility of TRANsCre-DIONE in the monkey brain suggests potential for future translational studies in traumatic brain injuries and neurodegenerative diseases.

## Supplementary information

Supplementary Information

Supplementary video 1

Supplementary video 2

## References

[1] Cregg, J. M. et al. Functional regeneration beyond the glial scar. *Exp Neurol.* **253**, 197–207 (2014). 10.1016/j.expneurol.2013.12.024 PMC3951813 24424280

[2] Su, Z., Niu, W., Liu, M. L., Zou, Y. & Zhang, C. L. In vivo conversion of astrocytes to neurons in the injured adult spinal cord. *Nat Commun.* **5**, 3338 (2014). 10.1038/ncomms4338 PMC3966078 24569435

[3] Ahuja, C. S. et al. Traumatic spinal cord injury-repair and regeneration. *Neurosurgery* **80**, S9–S22 (2017). 10.1093/neuros/nyw080 28350947

[4] Alizadeh, A., Dyck, S. M. & Karimi-Abdolrezaee, S. Traumatic spinal cord injury: an overview of pathophysiology, models and acute injury mechanisms. *Front Neurol.* **10**, 282 (2019). 10.3389/fneur.2019.00282 PMC6439316 30967837

[5] Assinck, P., Duncan, G. J., Hilton, B. J., Plemel, J. R. & Tetzlaff, W. Cell transplantation therapy for spinal cord injury. *Nat Neurosci.* **20**, 637–647 (2017). 10.1038/nn.4541 28440805

[6] Nagoshi, N. & Okano, H. iPSC-derived neural precursor cells: potential for cell transplantation therapy in spinal cord injury. *Cell Mol Life Sci.* **75**, 989–1000 (2018). 10.1007/s00018-017-2676-9 PMC11105708 28993834

[7] Yin, J. C. et al. Chemical conversion of human fetal astrocytes into neurons through modulation of multiple signaling pathways. *Stem Cell Rep* **12**, 488–501 (2019). 10.1016/j.stemcr.2019.01.003 PMC6409415 30745031

[8] Abad, M. et al. Reprogramming in vivo produces teratomas and iPS cells with totipotency features. *Nature.* **502**, 340–345 (2013). 10.1038/nature12586 24025773

[9] Li, H. & Chen, G. In vivo reprogramming for CNS repair: regenerating neurons from endogenous glial cells. *Neuron.* **91**, 728–738 (2016). 10.1016/j.neuron.2016.08.004 PMC5466364 27537482

[10] Xu, J., Du, Y. & Deng, H. Direct lineage reprogramming: strategies, mechanisms, and applications. *Cell Stem Cell.* **16**, 119–134 (2015). 10.1016/j.stem.2015.01.013 25658369

[11] Guo, Z. et al. In vivo direct reprogramming of reactive glial cells into functional neurons after brain injury and in an Alzheimer’s disease model. *Cell Stem Cell.* **14**, 188–202 (2014). 10.1016/j.stem.2013.12.001 PMC3967760 24360883

[12] Heinrich, C. et al. Directing astroglia from the cerebral cortex into subtype specific functional neurons. *PLoS Biol.* **8**, e1000373 (2010). 10.1371/journal.pbio.1000373 PMC2872647 20502524

[13] Matsuda, T. et al. Pioneer factor NeuroD1 rearranges transcriptional and epigenetic profiles to execute microglia-neuron conversion. *Neuron.* **101**, 472–485.e7 (2019). 10.1016/j.neuron.2018.12.010 30638745

[14] Takahashi, K. et al. Induction of pluripotent stem cells from adult human fibroblasts by defined factors. *Cell.* **131**, 861–872 (2007). 10.1016/j.cell.2007.11.019 18035408

[15] Victor, M. B. et al. Generation of human striatal neurons by microRNA-dependent direct conversion of fibroblasts. *Neuron.* **84**, 311–323 (2014). 10.1016/j.neuron.2014.10.016 PMC4223654 25374357

[16] Barres, B. A. New roles for glia. *J Neurosci.* **11**, 3685–3694 (1991). 10.1523/JNEUROSCI.11-12-03685.1991 PMC6575284 1720814

[17] De Pitta, M., Volman, V., Berry, H. & Ben-Jacob, E. A tale of two stories: astrocyte regulation of synaptic depression and facilitation. *PLoS Comput Biol.* **7**, e1002293 (2011). 10.1371/journal.pcbi.1002293 PMC3228793 22162957

[18] Chun, H. & Lee, C. J. Reactive astrocytes in Alzheimer’s disease: a double-edged sword. *Neurosci Res.* **126**, 44–52 (2018). 10.1016/j.neures.2017.11.012 29225140

[19] Okada, S., Hara, M., Kobayakawa, K., Matsumoto, Y. & Nakashima, Y. Astrocyte reactivity and astrogliosis after spinal cord injury. *Neurosci Res.* **126**, 39–43 (2018). 10.1016/j.neures.2017.10.004 29054466

[20] Anderson, M. A. et al. Astrocyte scar formation aids central nervous system axon regeneration. *Nature.* **532**, 195–200 (2016). 10.1038/nature17623 PMC5243141 27027288

[21] Burda, J. E. & Sofroniew, M. V. Reactive gliosis and the multicellular response to CNS damage and disease. *Neuron.* **81**, 229–248 (2014). 10.1016/j.neuron.2013.12.034 PMC3984950 24462092

[22] Chun, H. et al. Astrocytic proBDNF and tonic GABA distinguish active versus reactive astrocytes in hippocampus. *Exp Neurobiol.* **27**, 155–170 (2018). 10.5607/en.2018.27.3.155 PMC6050417 30022867

[23] Lee, H. Y. et al. Astrocytic monoamine oxidase B (MAOB)-gamma-aminobutyric acid (GABA) axis as a molecular brake on repair following spinal cord injury. *Signal Transduct Target Ther.* **10**, 295 (2025). 10.1038/s41392-025-02398-2 PMC12423301 40931022

[24] Brulet, R. et al. NEUROD1 instructs neuronal conversion in non-reactive astrocytes. *Stem Cell Rep* **8**, 1506–1515 (2017). 10.1016/j.stemcr.2017.04.013 PMC5470076 28506534

[25] Mattugini, N. et al. Inducing different neuronal subtypes from astrocytes in the injured mouse cerebral cortex. *Neuron.* **103**, 1086–1095.e5 (2019). 10.1016/j.neuron.2019.08.009 PMC6859713 31488328

[26] Brenner, M., Kisseberth, W. C., Su, Y., Besnard, F. & Messing, A. GFAP promoter directs astrocyte-specific expression in transgenic mice. *J Neurosci.* **14**, 1030–1037 (1994). 10.1523/JNEUROSCI.14-03-01030.1994 PMC6577554 8120611

[27] Park, Y. M., Chun, H., Shin, J. I. & Lee, C. J. Astrocyte specificity and coverage of hGFAP-CreERT2 [Tg(GFAP-Cre/ERT2)13Kdmc] mouse line in various brain regions. *Exp Neurobiol.* **27**, 508–525 (2018). 10.5607/en.2018.27.6.508 PMC6318562 30636902

[28] Liu, Y. et al. Glial fibrillary acidic protein-expressing neural progenitors give rise to immature neurons via early intermediate progenitors expressing both glial fibrillary acidic protein and neuronal markers in the adult hippocampus. *Neuroscience.* **166**, 241–251 (2010). 10.1016/j.neuroscience.2009.12.026 20026190

[29] Wu, Z. et al. Gene therapy conversion of striatal astrocytes into GABAergic neurons in mouse models of Huntington’s disease. *Nat Commun.* **11**, 1105 (2020). 10.1038/s41467-020-14855-3 PMC7046613 32107381

[30] Bi, F. et al. Reactive astrocytes secrete lcn2 to promote neuron death. *Proc Natl Acad Sci USA.* **110**, 4069–4074 (2013). 10.1073/pnas.1218497110 PMC3593910 23431168

[31] Zhao, M. L., Liu, J. S., He, D., Dickson, D. W. & Lee, S. C. Inducible nitric oxide synthase expression is selectively induced in astrocytes isolated from adult human brain. *Brain Res.* **813**, 402–405 (1998). 10.1016/s0006-8993(98)01023-3 9838203

[32] Walsh, K. A. et al. Antioxidant protection from HIV-1 gp120-induced neuroglial toxicity. *J Neuroinflamm* **1**, 8 (2004). 10.1186/1742-2094-1-8 PMC483061 15285794

[33] Hirrlinger, J. et al. Split-Cre complementation indicates coincident activity of different genes in vivo. *PLoS ONE* **4**, e4286 (2009). 10.1371/journal.pone.0004286 PMC2628726 19172189

[34] Hirrlinger, J. et al. Split-CreERT2: temporal control of DNA recombination mediated by split-Cre protein fragment complementation. *PLoS ONE* **4**, e8354 (2009). 10.1371/journal.pone.0008354 PMC2791205 20016782

[35] Tronche, F., Casanova, E., Turiault, M., Sahly, I. & Kellendonk, C. When reverse genetics meets physiology: the use of site-specific recombinases in mice. *FEBS Lett.* **529**, 116–121 (2002). 10.1016/s0014-5793(02)03266-0 12354622

[36] Wang, X. et al. The EF-1alpha promoter maintains high-level transgene expression from episomal vectors in transfected CHO-K1 cells. *J Cell Mol Med.* **21**, 3044–3054 (2017). 10.1111/jcmm.13216 PMC5661254 28557288

[37] Liu, M. L., Zang, T. & Zhang, C. L. Direct lineage reprogramming reveals disease-specific phenotypes of motor neurons from human ALS patients. *Cell Rep.* **14**, 115–128 (2016). 10.1016/j.celrep.2015.12.018 PMC4706770 26725112

[38] Seo, S., Lim, J. W., Yellajoshyula, D., Chang, L. W. & Kroll, K. L. Neurogenin and NeuroD direct transcriptional targets and their regulatory enhancers. *EMBO J.* **26**, 5093–5108 (2007). 10.1038/sj.emboj.7601923 PMC2140110 18007592

[39] Berninger, B. et al. Functional properties of neurons derived from in vitro reprogrammed postnatal astroglia. *J Neurosci.* **27**, 8654–8664 (2007). 10.1523/JNEUROSCI.1615-07.2007 PMC6672931 17687043

[40] Hulme, A. J., Maksour, S., St-Clair Glover, M., Miellet, S. & Dottori, M. Making neurons, made easy: the use of Neurogenin-2 in neuronal differentiation. *Stem Cell Rep* **17**, 14–34 (2022). 10.1016/j.stemcr.2021.11.015 PMC8758946 34971564

[41] Hu, X. et al. Region-restrict astrocytes exhibit heterogeneous susceptibility to neuronal reprogramming. *Stem Cell Rep* **12**, 290–304 (2019). 10.1016/j.stemcr.2018.12.017 PMC6373495 30713039

[42] Wang, L. L. et al. Revisiting astrocyte to neuron conversion with lineage tracing in vivo. *Cell.* **184**, 5465–5481.e16 (2021). 10.1016/j.cell.2021.09.005 PMC8526404 34582787

[43] Tang, Y., Liu, M. L., Zang, T. & Zhang, C. L. Direct reprogramming rather than iPSC-based reprogramming maintains aging hallmarks in human motor neurons. *Front Mol Neurosci.* **10**, 359 (2017). 10.3389/fnmol.2017.00359 PMC5676779 29163034

[44] Tepper, J. M., Tecuapetla, F., Koos, T. & Ibanez-Sandoval, O. Heterogeneity and diversity of striatal GABAergic interneurons. *Front Neuroanat.* **4**, 150 (2010). 10.3389/fnana.2010.00150 PMC3016690 21228905

[45] Liu, M. H. et al. Differential neuronal reprogramming induced by NeuroD1 from astrocytes in grey matter versus white matter. *Neural Regen Res.* **15**, 342–351 (2020). 10.4103/1673-5374.265185 PMC6905344 31552908

[46] An, H. et al. Differential cellular tropism of lentivirus and adeno-associated virus in the brain of cynomolgus monkey. *Exp Neurobiol.* **25**, 48–54 (2016). 10.5607/en.2016.25.1.48 PMC4766114 26924933

[47] Park, Y. G. et al. Protection of tissue physicochemical properties using polyfunctional crosslinkers. *Nat Biotechnol.* **37**, 73–83 (2019). 10.1038/nbt.4281 PMC6579717 30556815

[48] Cho, H. H. et al. Isl1 directly controls a cholinergic neuronal identity in the developing forebrain and spinal cord by forming cell type-specific complexes. *PLoS Genet.* **10**, e1004280 (2014). 10.1371/journal.pgen.1004280 PMC3998908 24763339

[49] Russo, A. et al. Pharmacological evaluation of intravenous delivery of 5-bromodeoxyuridine to patients with brain tumors. *Cancer Res.* **44**, 1702–1705 (1984). 6704976

[50] Roybon, L. et al. The origin, development and molecular diversity of rodent olfactory bulb glutamatergic neurons distinguished by expression of transcription factor NeuroD1. *PLoS ONE* **10**, e0128035 (2015). 10.1371/journal.pone.0128035 PMC4451148 26030886

[51] Zhou, H. et al. Glia-to-neuron conversion by CRISPR–CasRx alleviates symptoms of neurological disease in mice. *Cell.* **181**, 590–603.e16 (2020). 10.1016/j.cell.2020.03.024 32272060

[52] Qian, H. et al. Reversing a model of Parkinson’s disease with in situ converted nigral neurons. *Nature.* **582**, 550–556 (2020). 10.1038/s41586-020-2388-4 PMC7521455 32581380

[53] Kijima, K. et al. The acute phase serum zinc concentration is a reliable biomarker for predicting the functional outcome after spinal cord injury. *eBioMedicine.* **41**, 659–669 (2019). 10.1016/j.ebiom.2019.03.003 PMC6444130 30902739

