# Supplementary material for: TRANsCre-DIONE transdifferentiates scar-forming reactive astrocytes into functional motor neurons
Source: Exp Mol Med. 2026 Sep 11;58(9):3158–76. doi: 10.1038/s12276-026-01815-y (PMC13633286; doi:10.1038/s12276-026-01815-y)
Supplement: Supplementary file 1 [file 12276_2026_1815_MOESM1_ESM.pdf]

Supplementary Information

# TRANsCre-DIONE transdifferentiates scar-forming reactive astrocytes into functional motor neurons

Heeyoung An, Hye-Lan Lee, Doo-Wan Cho, Jinpyo Hong, Hye Yeong Lee, Jung Moo Lee, SunYeong Choi, In-Young Hwang, Junsung Woo, Jaekwang Lee, MinGu Park, Young-Su Yang, Su-Cheol Han, Yoon Ha, C. Justin Lee

**Supplementary Fig. S1**

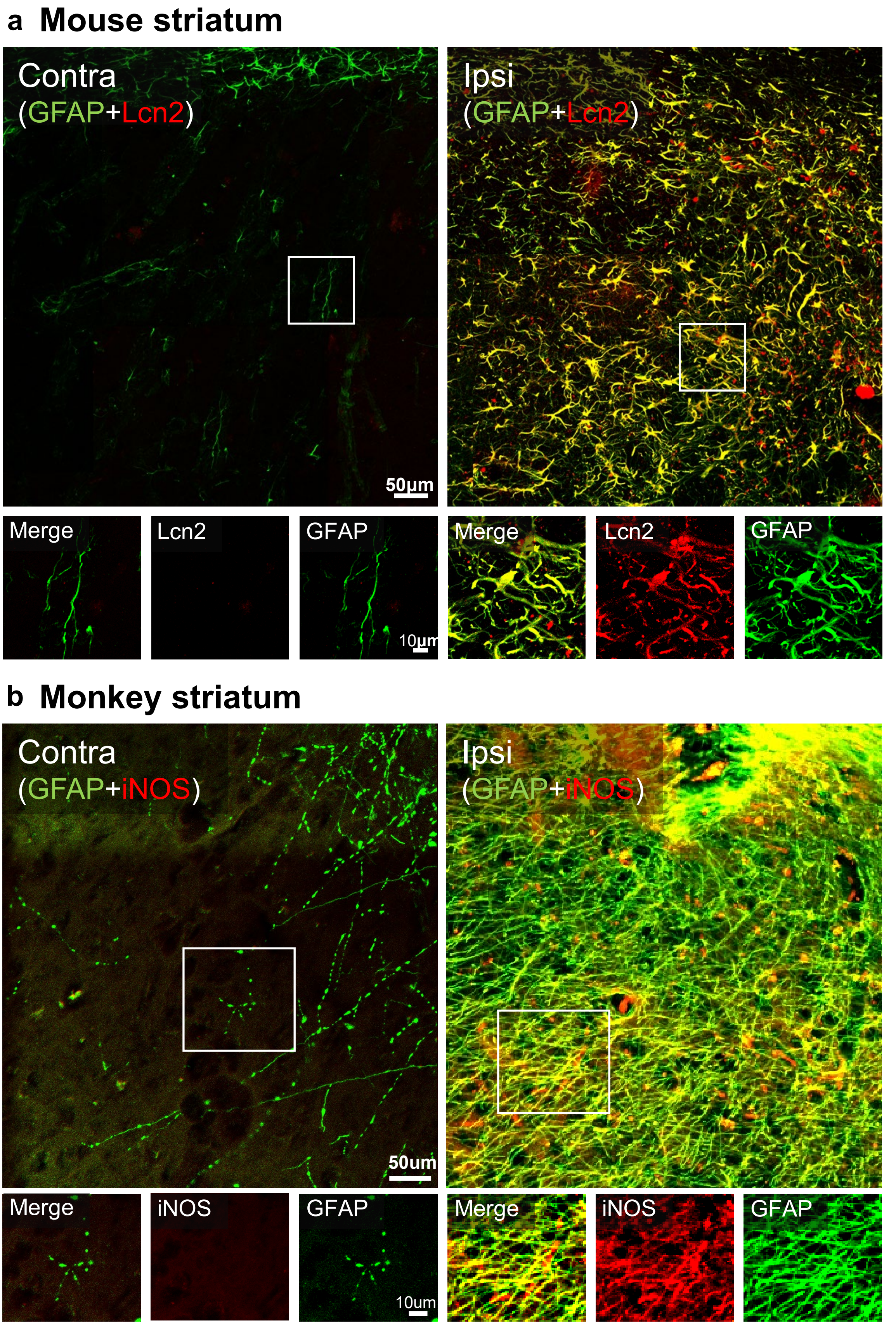


**Supplementary Fig. S1. Lcn2 or iNOS with GFAP expression in contra or ipsi lateral hemispheres, related with Fig. 1**

(a) Colocalization of Lcn2 (red) and GFAP (green) in contra lateral (left) and ipsi lateral (right) striatum of mouse. Lower panel shows magnified images. Scale bar = 50 µm, 10 µm (magnified images) (b) Colocalization of iNOS (red) and GFAP (green) in contra lateral (left) and ipsi lateral (right) putamen of cynomolgus monkey. Lower panel shows magnified images. Scale bar = 50 µm, 10 µm (magnified images).

# Supplementary Fig. S2

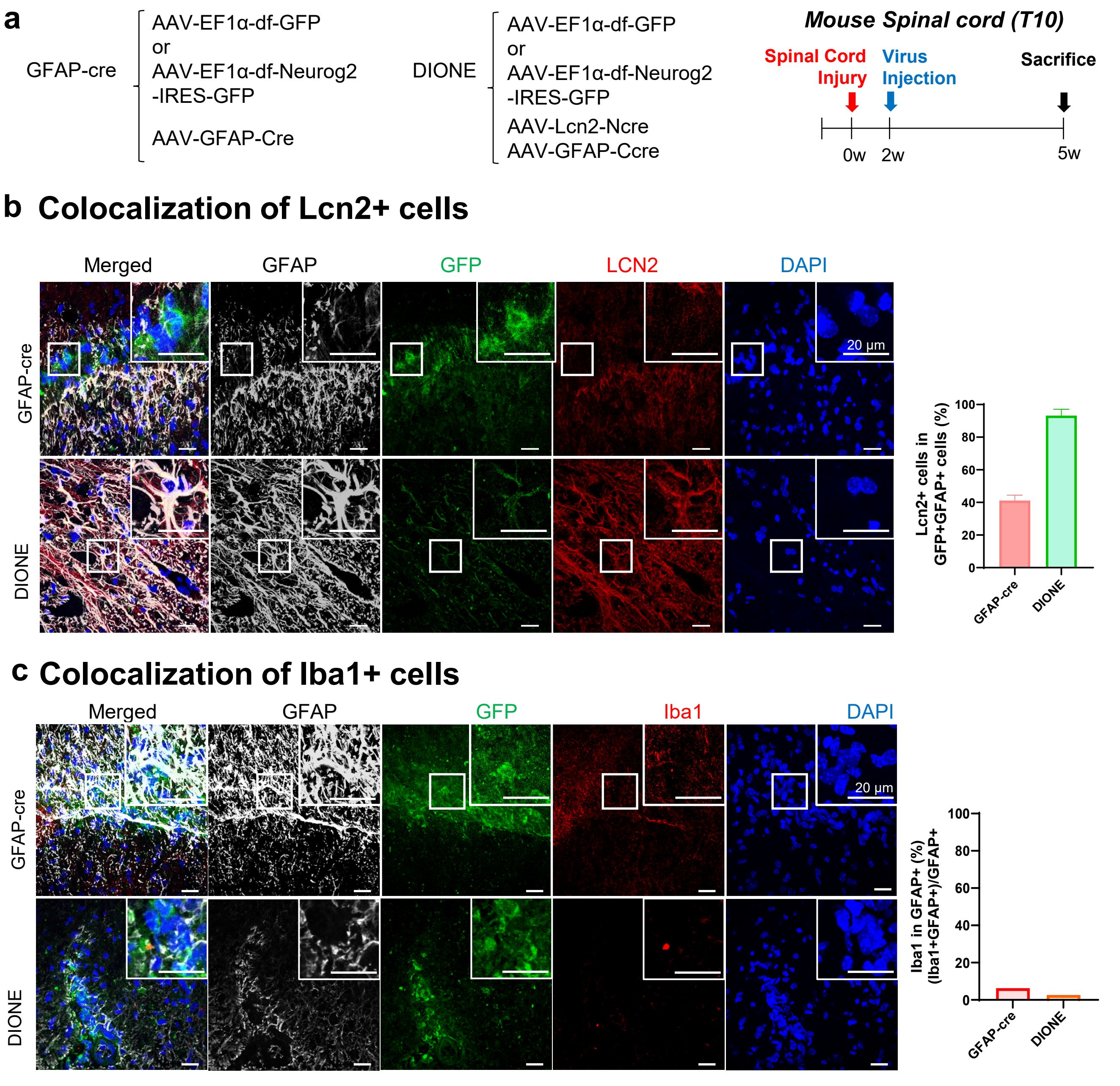


**Supplementary Fig. S2. Cell-type specific expression of TRANsCre-DIONE, related with Fig. 1**

(a) Schematic of the experimental timeline and viral injection strategy into the T10 spinal cord following traumatic injury. (b) Representative immunofluorescence images colocalization of Lcn2 (red) and GFP expression (green), and GFAP (gray) with DAPI (blue) in the perilesional area of the spinal cord driven by the GFAP-Cre or DIONE system. The summary bar graph shows the percentage of LCN2+GFAP+GFP+ cells in GFP+GFAP+ population (%). (c) Representative immunofluorescence images of Iba1 (red), GFAP (gray), and GFP expression (green), and DAPI (blue). The summary bar graph shows the percentage of Iba1+GFAP+GFP+ population among GFAP+GFP+ cells (%) to verify the specificity of the DIONE system against microglial expression.

# Supplementary Fig. S3

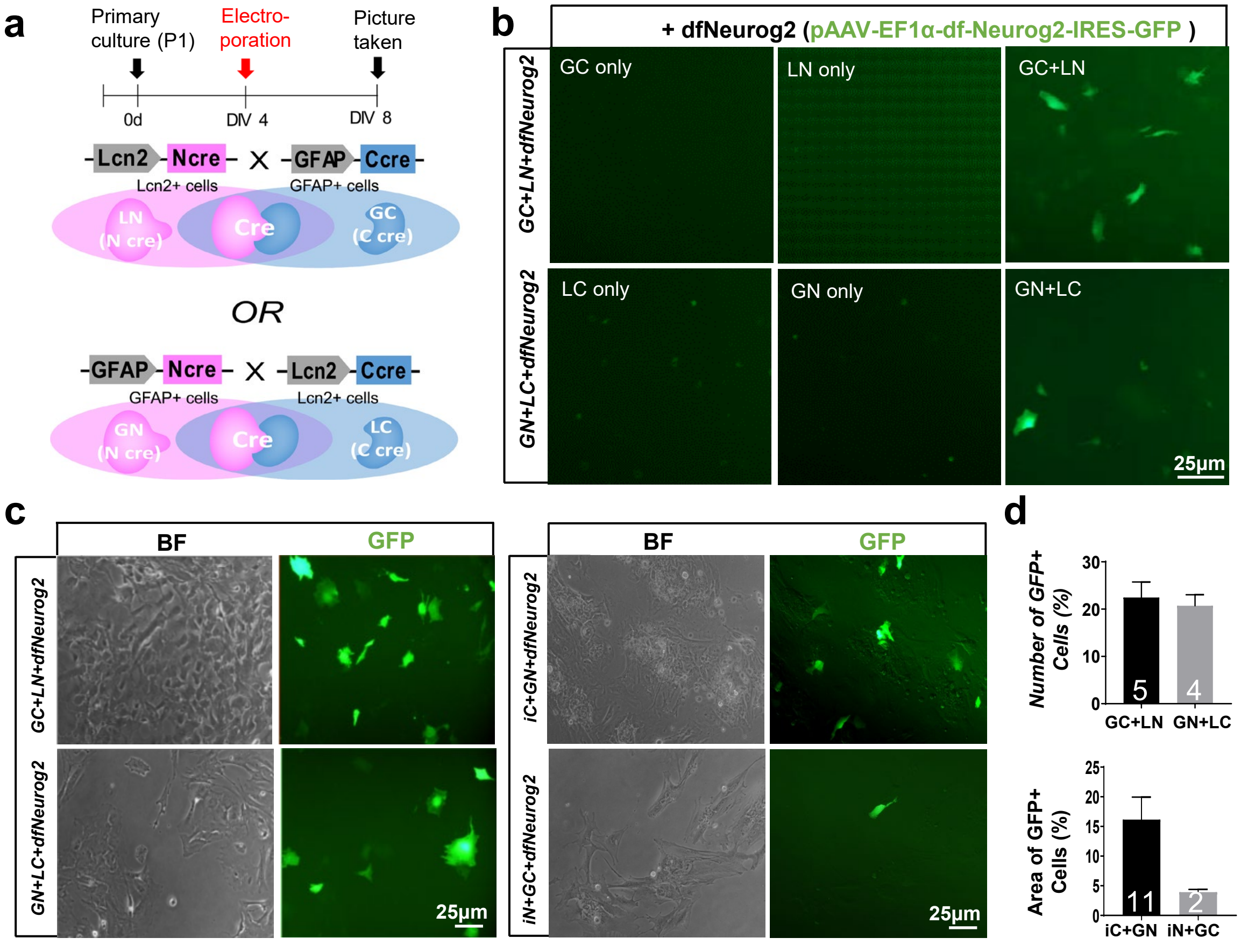


**Supplementary Fig. S3. Cell-specificity and expression-efficiency of Split-Cre, related with Fig. 1**

(a) Experimental protocol for working test of the split-Cre system in primary cultured astrocytes. Electroporation was performed DIV 4 and the fluorescence was detected DIV 8 (4 days after electroporation). (b) pAAV-EF1α-df-Ngn2-IRES-GFP (dfNgn2; GFP) was electroporated with Ccre (GC, GFAP-Ccre; LC, Lcn2-Ccre) or Ncre (LN, Lcn2-Ncre; GN, GFAP-Ncre) only or both. Scale bar = 25 μm. (c) Efficiency test for expression of each pair of split-Cre with dfNgn2. GFAP and Lcn2 promoters, the pair of GC+LN and GN+LC (left). iNOS and GFAP promoters, the pair of iC+GN and iN+GC (right). Bright field (BF) images show cell density, GFP fluorescence (GFP) shows the gene expression. Scale bar = 25 μm. (d) Summary bar graphs showing the percentage of the GFP-expressing cells or area in the fields of views. n = 5 (GC+LN), 4 (GN+LC). n = 11 (iC+GN), 2 (iN+GC).

# Supplementary Fig. S4

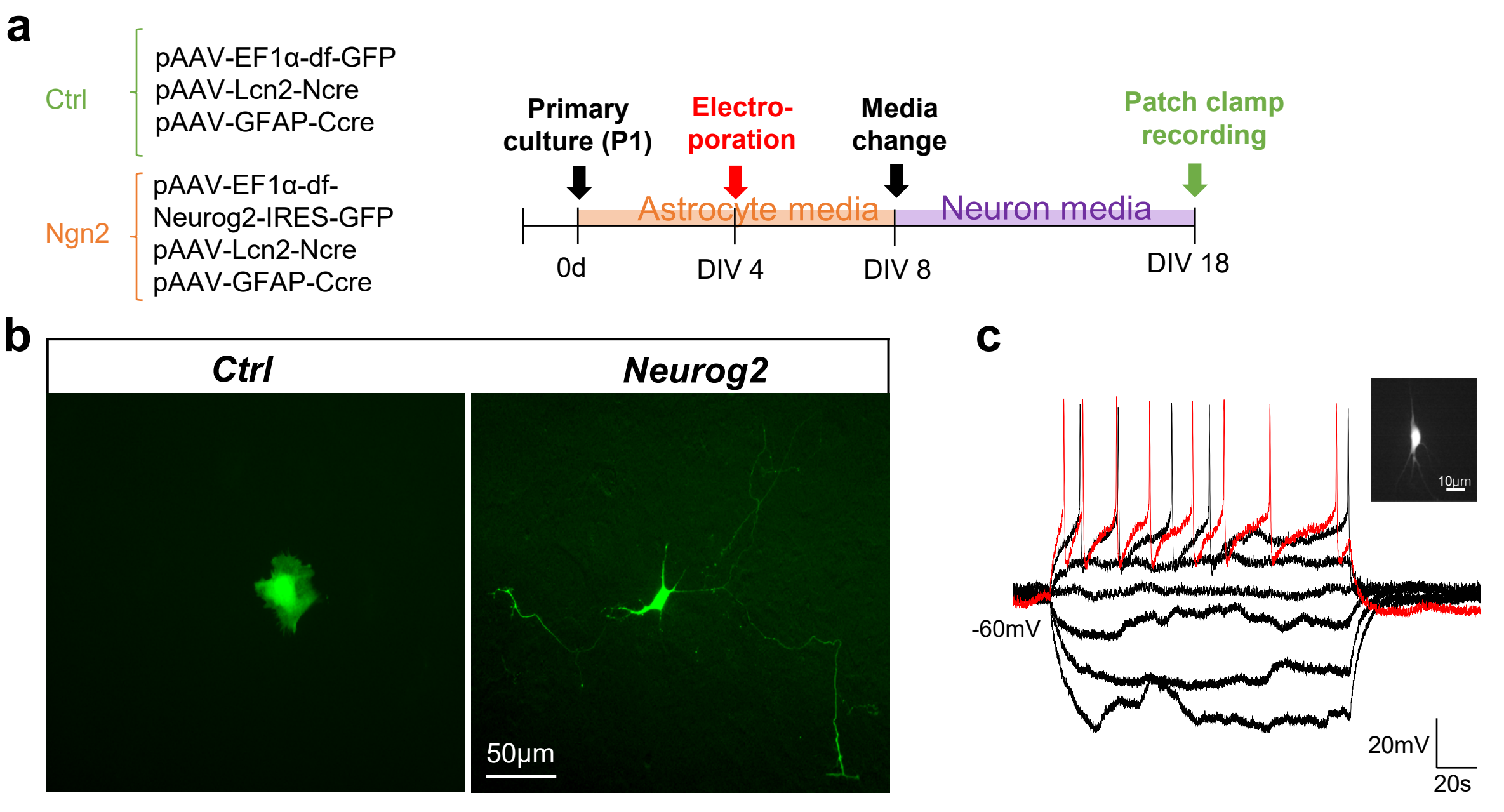


**Supplementary Fig. S4. Transdifferentiation of reactive astrocytes into functional neurons in culture, related with Fig. 2**

(a) A mixture of 3 vectors (TRANsCre-DIONE, 5 µg each) were electroporated in primary cultured astrocytes for transdifferentiation of reactive astrocytes into neurons and the timeline of transfection and treating neuronal media. Electroporation was performed at days *in vitro* (DIV) 4, media was changed to neuronal media 4 days after electroporation in Neurog2 group. The transdifferentiated cells were recorded by whole-cell patch-clamp recording at DIV 18. (b) Transdifferentiated cells (GFP) show the neuron-like morphology 14 days after electroporation (DIV 18) in Neurog2 group. Scale bar = 50µm. (c) Whole-cell patch clamp recording with an GFP expressing cell (inset, scale bar = 10µm) was performed at DIV 18. Neuron-like cell shows action potentials, but not spontaneous. Scale bar = 20 mV, 20 sec.

## Supplementary Fig. S5

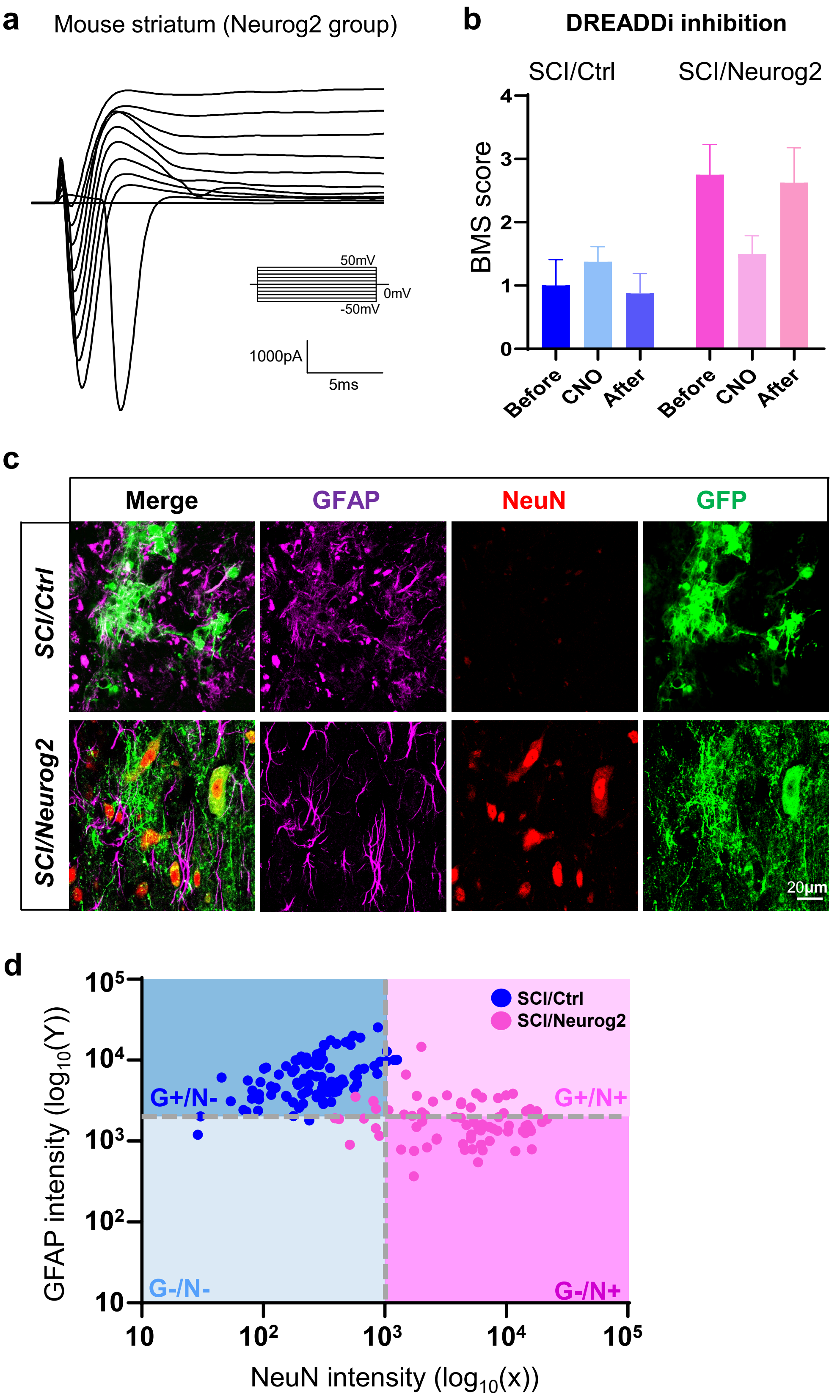


**Supplementary Fig. S5. Transdifferentiated functional neurons in mouse striatum and the efficiency of transdifferentiation in SCI, related with Figs. 2 and 4**

(a) Na-K currents were recorded by voltage-clamp step protocol in GFP-expressing cells in mouse striatum. (b) Comparison of BMS score after chemogenetic inhibition between SCI/Ctrl (N = 4) and SCI/Neurog2 (N = 3) group. (c) Colocalization of GFAP (magenta), NeuN (red) and GFP (green) in SCI/Ctrl and SCI/Neurog2 groups. Scale bar = 20 μm. (d) Scatter plot of GFP-expressing cells classified by GFAP and NeuN intensity from SCI/Ctrl (blue,) and SCI/Neurog2 (pink,) groups. GFP+ cells were divided in four groups, G+/N- (blue), G-/N- (light blue), G+/N+ (light pink), G-/N+ (pink). n =102 (SCI/Ctrl), 77 (SCI/Neurog2).

## Supplementary Fig. S6

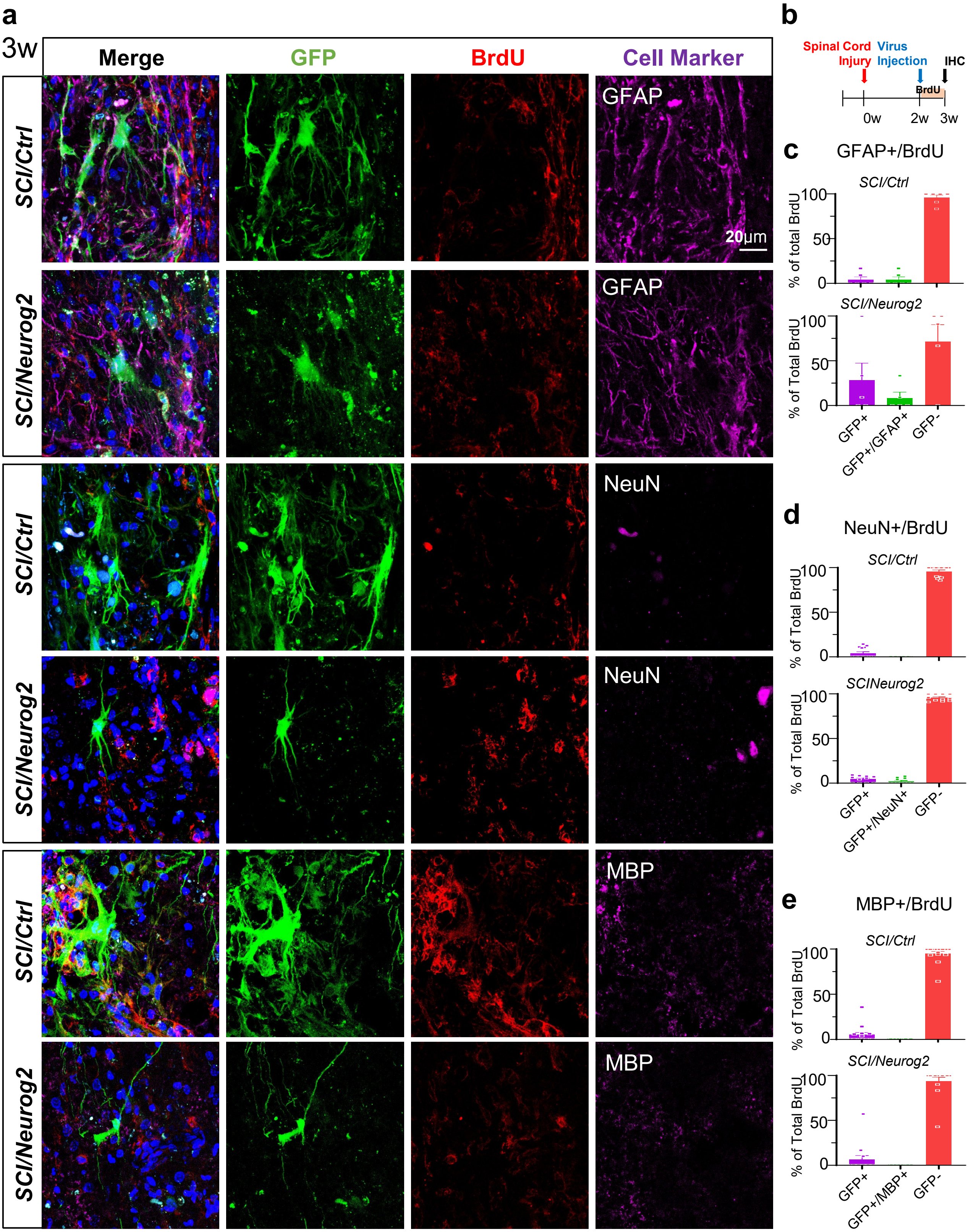


**Supplementary Fig. S6. Transdifferentiated neurons are mostly originated from non-proliferating cells, related with Fig. 8**

(a) Colocalization of BrdU (red, treated for 1week from 2-3 weeks after injury) in GFP-expressing cells (green) with cell markers (magenta; GFAP, upper; NeuN, middle; MBP, lower) in SCI/Ctrl and SCI/Neurog2 groups. The mice were sacrifice 3 weeks after injury (3w). Scale bar = 20 μm. (b) Timeline of treating BrdU and IHC after sacrificing in SCI mice. (c) Scattered bar graph showing comparison of percentage of BrdU-positive cells in GFP+, GFAP+/GFP+ and GFP- cells in SCI/Ctrl and SCI/Neurog2 groups. n = 7 (SCI/Ctrl group), 17 (SCI/Neurog2 group). (d) Scattered bar graph showing comparison of percentage of BrdU-positive cells in GFP+, NeuN+/GFP+ and GFP- cells in SCI/Ctrl and SCI/Neurog2 groups. n = 7 (SCI/Ctrl group), 17 (SCI/Neurog2 group). (e) Scattered bar graph showing comparison of percentage of BrdU-positive cells in GFP+, MBP+/GFP+ and GFP- cells in SCI/Ctrl and SCI/Neurog2 groups. n = 7 (SCI/Ctrl group), 17

## Supplementary Fig. S7

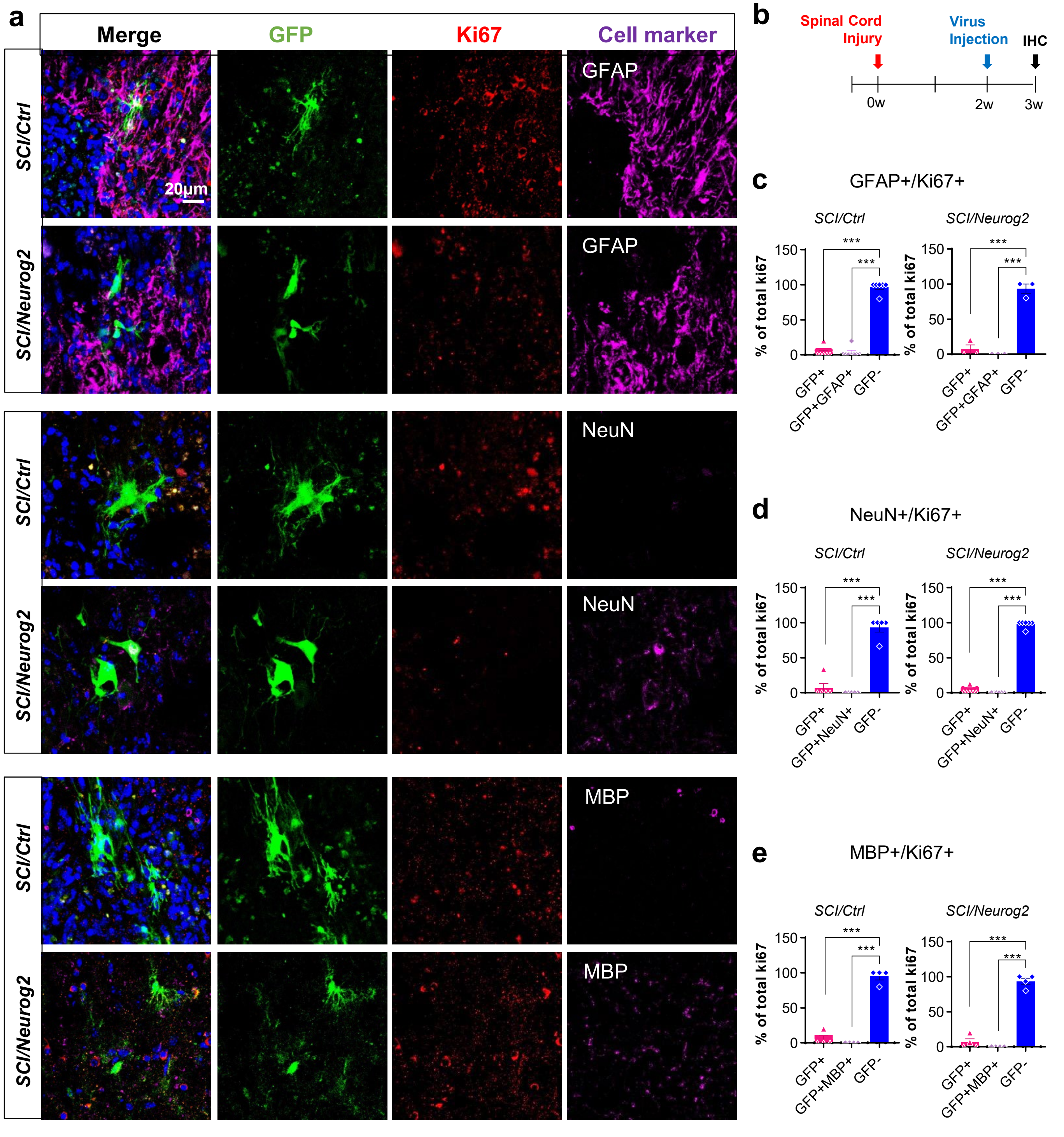


**Supplementary Fig. S7. Transdifferentiated neurons are mostly originated from non-proliferating cells with Ki67 labeling in the early stage, related with Fig. 8**

(a) Colocalization of Ki67 (red) in GFP-expressing cells (green) with cell markers (magenta; GFAP, upper; NeuN, middle; MBP, lower) in SCI/Ctrl and SCI/Neurog2 groups. The mice were sacrifice 3 weeks after injury (3w). Scale bar = 20 μm. (b) Timeline of immunostaining after sacrificing in SCI mice. (c) Scattered bar graph showing comparison of percentage of Ki67-positive cells in GFP+, GFAP+/GFP+ and GFP- cells in SCI/Ctrl and SCI/Neurog2 groups. (d) Scattered bar graph showing comparison of percentage of Ki67-positive cells in GFP+, NeuN+/GFP+ and GFP- cells in SCI/Ctrl and SCI/Neurog2 groups. (e) Scattered bar graph showing comparison of percentage of Ki67-positive cells in GFP+, MBP+/GFP+ and GFP- cells in SCI/Ctrl and SCI/Neurog2 groups. n =7 (SCI/Ctrl group), 17 (SCI/Neurog2 group).

# Supplementary Fig. S8

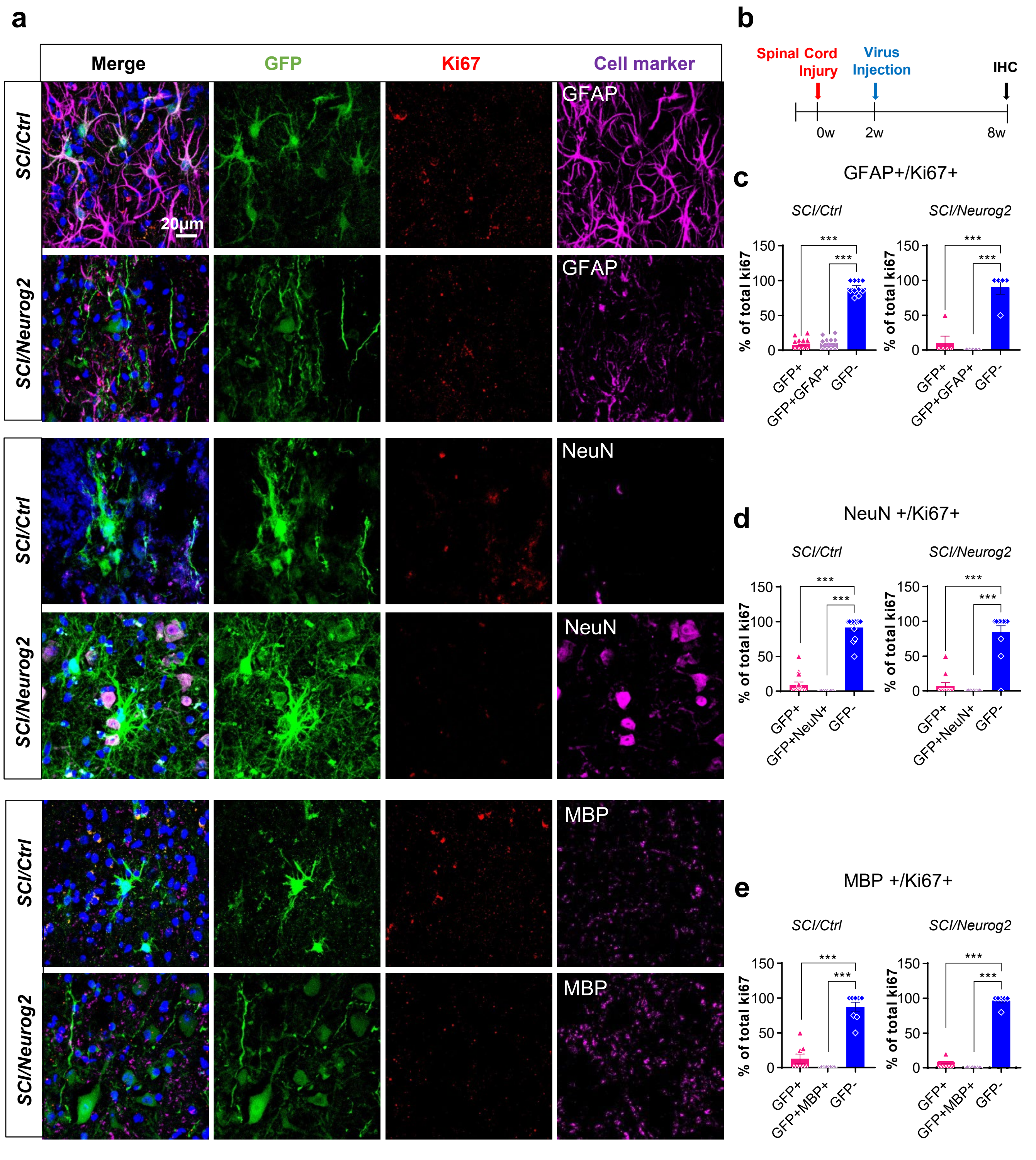


**Supplementary Fig. S8. Transdifferentiated neurons are mostly originated from non-proliferating cells with Ki67 labeling in the late stage, related with Fig. 8**

(a) Colocalization of Ki67 (red) in GFP-expressing cells (green) with cell markers (magenta; GFAP, upper; NeuN, middle; MBP, lower) in SCI/Ctrl and SCI/Neurog2 groups. The mice were sacrifice 8 weeks after injury (8w). Scale bar = 20 μm. (b) Timeline of the immunostaining after sacrificing in SCI mice. (c) Scattered bar graph showing comparison of percentage of Ki67-positive cells in GFP+, GFAP+/GFP+ and GFP- cells in SCI/Ctrl and SCI/Neurog2 groups. (d) Scattered bar graph showing comparison of percentage of Ki67-positive cells in GFP+, NeuN+/GFP+ and GFP- cells in SCI/Ctrl and SCI/Neurog2 groups. (e) Scattered bar graph showing comparison of percentage of Ki67-positive cells in GFP+, MBP+/GFP+ and GFP- cells in SCI/Ctrl and SCI/Neurog2 groups. n=8 (SCI/Ctrl group), 8 (SCI/Neurog2 group).

## Supplementary Fig. S9

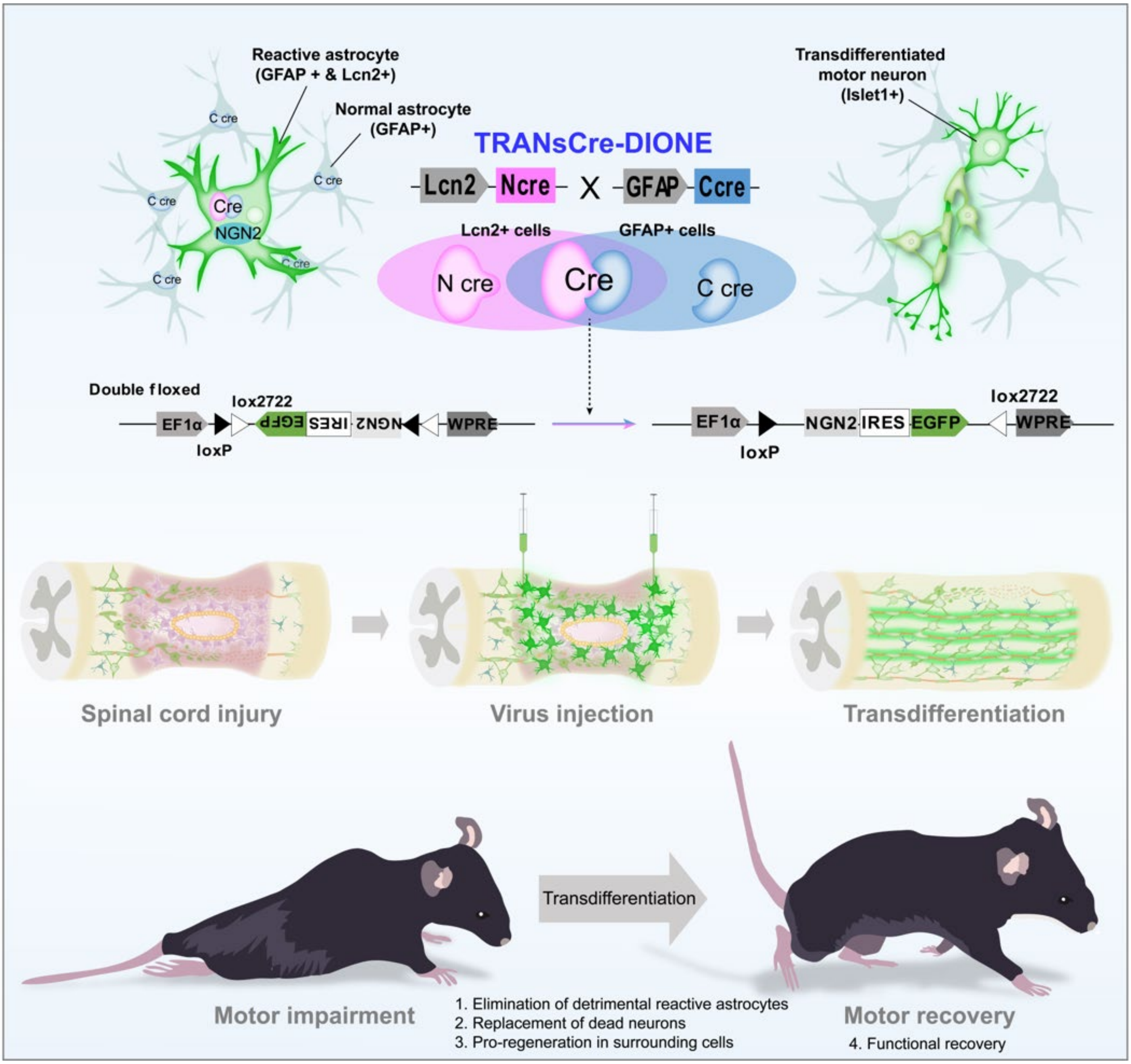


**Supplementary Fig. S9. Schematic principle of treating SCI via direct reprogramming of reactive astrocytes into motor neurons.**
